# Choroid plexus enlargement associates with serum neurofilament and predicts relapse-free progression in multiple sclerosis

**DOI:** 10.1016/j.xcrm.2026.102609

**Published:** 2026-02-09

**Authors:** Vinzenz Fleischer, Muriel Schraad, Gabriel Gonzalez-Escamilla, Ruth Schneider, Tobias Brummer, Falk Steffen, Maria Protopapa, Nicholas Hanuscheck, Anke Salmen, Sven G. Meuth, Felix Luessi, Heinz Wiendl, Luisa Klotz, Ralf Gold, Carsten Lukas, Stefan Bittner, Sergiu Groppa, Frauke Zipp

**Affiliations:** 1 Department of Neurology, Research Center for Immunotherapy (FZI) and Focus Program Translational Neuroscience (FTN), Rhine Main Neuroscience Network (rmn2), University Medical Center of the Johannes Gutenberg University Mainz, 55131 Mainz, Germany; 2 Department of Neurology, St. Josef-Hospital, Ruhr-University Bochum, 44791 Bochum, Germany; 3 Department of Neurology, University Hospital Düsseldorf, Medical Faculty, Heinrich Heine University, 40225 Düsseldorf, Germany; 4 Department of Neurology and Clinical Neuroscience, Faculty of Medicine, Medical Center University of Freiburg, 79106 Freiburg, Germany; 5 Department of Neurology, University Hospital Münster, Westfälische-Wilhelms-University Münster, 48149 Münster, Germany; 6 Institute of Neuroradiology, St. Josef Hospital, Ruhr University Bochum, 44791 Bochum, Germany

**Keywords:** multiple sclerosis, choroid plexus, neurofilament light chain, disease progression, magnetic resonance imaging, neurodegeneration, progression independent of relapse activity, neuroinflammation

## Abstract

The choroid plexus (CP), a key regulator of cerebrospinal fluid production and immune cell trafficking, is increasingly recognized as a potential magnetic resonance imaging (MRI) biomarker in multiple sclerosis (MS). Serum neurofilament light chain (sNfL) serves as a sensitive blood-based indicator of neuroaxonal injury. We investigate the prognostic value of CP volume and its longitudinal change for neurodegeneration, defined by sNfL levels and disability progression. In a prospective, multicenter, longitudinal study, 891 people with MS undergo high-resolution 3T MRI, sNfL measurement, and clinical assessment over 6 years; 434 meet criteria for inclusion. CP volume correlates with sNfL after adjustment for demographic, clinical, treatment, and imaging variables. In a 2-year MRI subcohort (*n* = 209), CP enlargement likewise associates with sNfL. High CP volume confers a 1.8-fold increased risk of disability worsening and a 2.7-fold increased risk of progression independent of relapse activity. These findings identify CP imaging as a promising non-invasive biomarker of neuroaxonal loss and relapse-free progression.

## Highlights

• Choroid plexus (CP) volume associates with serum neurofilament levels in MS
• CP enlargement over time reflects ongoing neuroaxonal injury
• High CP volume predicts relapse-free progression in people with MS

## Graphical abstract

**Figure:**
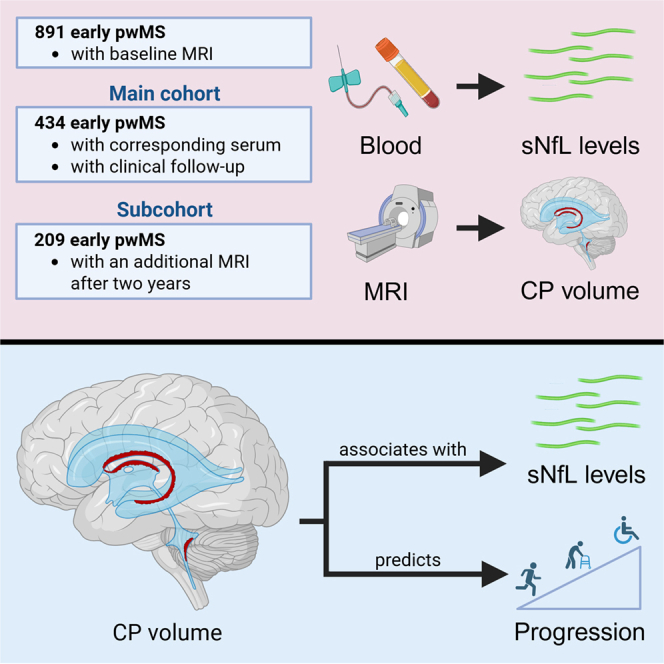

## Teaser

In this multicenter longitudinal study, Fleischer et al. show that enlargement of the choroid plexus, a key gateway between the blood and cerebrospinal fluid, signals early neuroaxonal injury and predicts silent progression in MS. Their findings suggest that volumetric changes at this barrier contribute to neurodegeneration and clinical disability in MS.

## Introduction

Multiple sclerosis (MS) is characterized by neuroinflammation, demyelination, and neuroaxonal damage.^1^ Neuroimaging studies have been instrumental in elucidating the pathological processes underlying MS, with magnetic resonance imaging (MRI) serving as a cornerstone for diagnosis and monitoring.

Compelling evidence supports an essential role of the choroid plexus (CP) as a key invasion route for immune cells into the CNS and thus as part of immune surveillance.^2^ The CP separates the cerebrospinal fluid (CSF) from blood and serves as an immunological niche.^3^ In MS, immune cells invading the ventricles have to eventually pass the CP epithelium.^2^ Histopathologic findings demonstrated an enlargement of the CP in MS due to edema with high concentrations of T lymphocytes, dendritic cells, and activated macrophages.^4^ On the macroscale, CP enlargement was confirmed with advanced imaging algorithms in high-resolution MRI in people with MS (pwMS).^5^ Greater CP volume was associated with increased lesion activity and greater brain atrophy,^6^^,^^7^ suggesting that CP enlargement promotes the inflammatory part of the pathology.

Therefore, we aimed to explore the association between CP enlargement and serum neurofilament light (sNfL) levels, as a marker of neuroaxonal loss, in a large longitudinal multicenter cohort of pwMS early in the disease course and to find out whether CP volume has prognostic value for future disability worsening. In this report, we found that increased CP volume associated with early neuroaxonal loss and predicted the development of silent disease progression over time, suggesting that the CP is very early, dynamically as well as sustainably involved in MS neuronal compartment pathology.

## Results

### Study cohort characteristics

An overview of the demographics and clinical characteristics of the main cohort and subcohort is depicted in Table 1.

**Table 1:** Basic data

	Main cohort (*n* = 434)	Subcohort (*n* = 209)
Sex (female/male)	310/124	149/60
Age at MRI, mean (±SD) (years)	38.7 ± 12.0	42.9 ± 12.4
Disease duration, mean (±SD) (years)	1.6 ± 3.2	2.6 ± 4.2
EDSS score, median (range) (at baseline)	1.5 (0–7.5)	1.0 (0–7.5)
EDSS score, median (range) (at follow-up)	–	1.5 (0–7.5)
DMT baseline (none/platform/high efficacy)	271/129/34	56/121/32
DMT follow-up (none/platform/high efficacy)	–	32/128/49
Duration of MRI follow-up, mean (±SD) (years)	–	2.4 ± 1.3
Serum NfL, mean (±SD) (pg/mL)	17.4 ± 27.3	17.0 ± 31.6
Serum NfL follow-up, mean (±SD) (pg/mL)	–	10.2 ± 15.7
CP, mean (±SD) (at baseline; mm^3^)	1,066.3 ± 488.9	1,078.3 ± 573.3
CP, mean (±SD) (at follow-up; mm^3^)	–	1,111.4 ± 569.5
Change of plexus volume, mean (±SD) (mm^3^)	–	33.1 ± 197.6

Demographical and clinical data as well as MRI (CP volume) and blood-based (sNfL levels) measures of people with MS (pwMS) in the main cohort (*n* = 434) and the subcohort (*n* = 209). CP, choroid plexus; EDSS, Expanded Disability Status Scale; DMT, disease-modifying treatment; MRI, magnetic resonance imaging; SD, standard deviation; sNfL, serum neurofilament.

**Figure 1:**
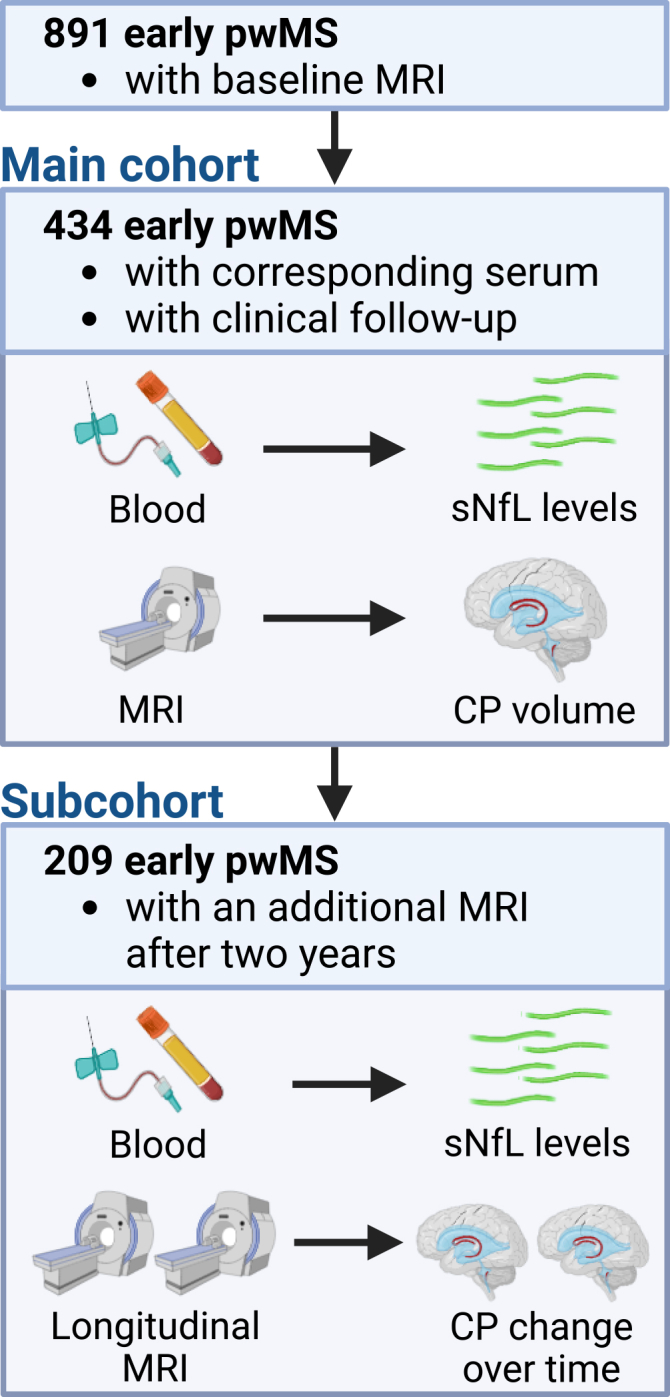
Study design 891 early pwMS (time since diagnosis <5 years) underwent baseline high-resolution MRI with standardized protocol in all centers. Out of these, 434 pwMS had serum available for sNfL measurement. In a subcohort of 209 pwMS, a subsequent MRI was performed after 2 years of follow-up.

In total, 891 pwMS with standardized 3 Tesla MRI were screened; 434 patients fulfilled the imaging requirements for CP volume measurement, had serum available at the imaging time point, and were enrolled in the main study. Figure 1 outlines our study design. The mean (±standard deviation [SD]) age of the included 434 pwMS was 38.7 ± 12.0 years; 310 (71.4%) patients were female and 124 (28.6%) were male. Mean disease duration at MRI and serum acquisition was 1.6 ± 3.2 years; median disability (quantified by Expanded Disability Status Scale [EDSS]) was 1.0 (range 0–7.5). Thirty-four pwMS were on high-efficacy disease-modifying treatment (DMT; natalizumab, alemtuzumab, anti-CD20 antibodies, or sphingosine-1-phosphate receptor modulators).

In the subcohort with an additional MRI after 2 years (*n* = 209), the mean (±SD) age was 42.9 ± 12.4 years; 149 (71.3%) patients were female and 60 (28.7%) were male. Mean disease duration at baseline MRI was 2.6 ± 4.2 years; median disability (quantified by EDSS) was 1.0 (range 0–7.5). Thirty-two pwMS were on high-efficacy DMT.

### Association of CP volume with sNfL levels and the predictive capacity of CP volume for disability accumulation in MS

**Figure 2:**
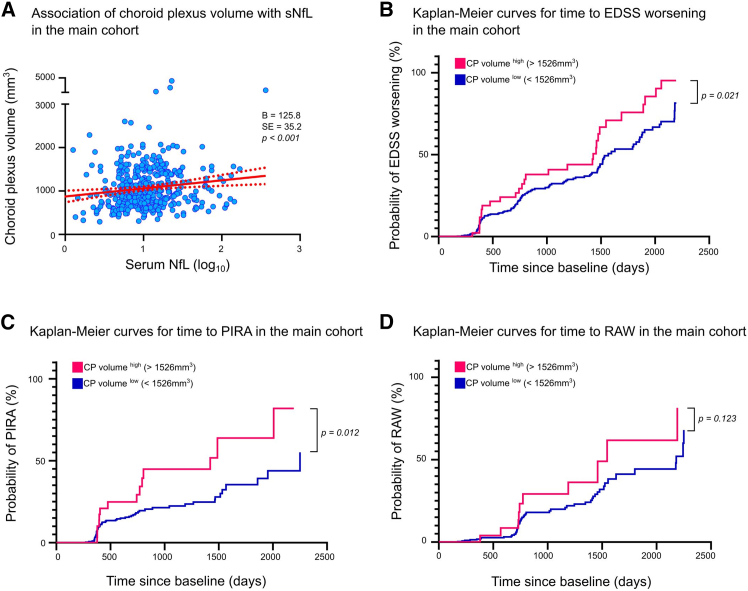
Association of CP volume with sNfL levels and the predictive capacity of CP volume for disability accumulation in MS (A) Association of choroid plexus (CP) volume with sNfL levels (log_10_) at baseline in the main cohort. Linear fit (red) to individual values from the main cohort (blue) is shown with 95% CI (dotted red). The regression model was corrected for sex, age, disease duration, Expanded Disability Status Scale (EDSS) scores, disease-modifying treatment, intracranial volume, scanner, lateral ventricle volume, T2 lesion volume, gadolinium-enhancing lesions, and body mass index. (B–D) Kaplan-Meier curves to predict time to (B) EDSS worsening (CP volume^low^ = 422 and CP volume^high^ = 46), (C) progression independent of relapse activity (PIRA) (CP volume^low^ = 317 and CP volume^high^ = 36), and (D) relapse-associated worsening (RAW) (CP volume^low^ = 300 and CP volume^high^ = 31). Dichotomized groups were based on the optimized cutoff values of CP volume derived from a receiver operating characteristics analysis (STAR Methods). CI, confidence interval; B, regression coefficient; SE, standard error; p, *p* value.

Overall, the results from the main cohort demonstrated a correlation of CP volume with sNfL levels in pwMS in the regression model correcting for sex, age, disease duration, EDSS score, DMT, intracranial volume, scanner, lateral ventricle volume, T2 lesion volume, gadolinium-enhancing lesions, and BMI (Figure 2A) (B = 125.8; SE = 35.2; *p* < 0.001). Significant influential covariates in the main cohort were age, lateral ventricle volume, and T2 lesion volume (Table S2).

Kaplan-Meier analysis using dichotomized CP volume was applied to predict time to disability worsening. Dichotomized patient groups were based on the optimized cutoff values of CP volume. The associated criterion of the Youden index was a CP volume of 1,526 mm^3^ (CP volume < 1,526 mm^3^ = “CP volume^low^” and CP volume > 1,526 mm^3^ = “CP volume^high^”). Thereby, pwMS with a CP volume^high^ revealed a 1.8-fold increased risk of EDSS worsening compared to CP volume^low^ at baseline (95% confidence interval [CI] = 1.087–2.812; *p* = 0.021; Figure 2B). We then applied Kaplan-Meier analysis to predict PIRA (progression independent of relapse activity) or RAW (relapse-associated worsening) versus patients who did not attain PIRA or RAW. PIRA was defined as an increase in EDSS score that occurred in the absence of a clinical relapse, while RAW was defined as an increase in EDSS score that occurred after a documented clinical relapse. CP volume^high^ revealed a 2.7-fold risk of PIRA compared to CP volume^low^ (95% CI = 1.240–5.649; *p* = 0.012; Figure 2C), while CP volume was not significantly different in the time-to-event analysis of RAW (*p* = 0.123; Figure 2D). In an additional multivariate Cox regression analysis adjusting for sNfL levels, CP volume remained a significant predictor of increased risk for PIRA (hazard ratio = 1.95; 95% CI = 1.05–3.62; *p* = 0.035).

### Association of CP volume with sNfL levels in the subcohort with longitudinal MRI

**Figure 3:**
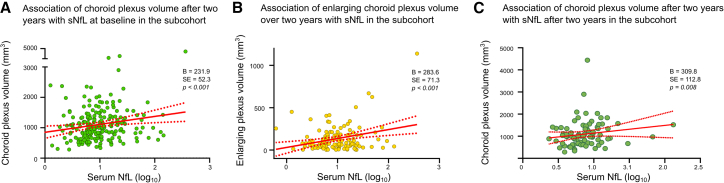
Association of CP volume with sNfL levels in the subcohort with longitudinal MRI (A) Association of CP volume after 2 years of MRI follow-up with sNfL levels (log_10_). (B) Association of enlarging CP volume with sNfL levels (log_10_). (C) Association of CP volume after 2 years with sNfL levels (log_10_) after 2 years. The regression models were corrected for sex, age, disease duration, Expanded Disability Status Scale (EDSS) scores, disease-modifying treatment, intracranial volume, lateral ventricle volume, number of relapses between MRIs, time between MRIs, T2 lesion volume, gadolinium-enhancing lesions, and BMI. Linear fit (red) to individual values is shown with 95% CI (dotted red). CI, confidence interval; B, regression coefficient; SE, standard error; p, *p* value.

In those pwMS with a follow-up MRI acquired using the same protocol and scanner as at baseline, mean CP volume increased over 2 years (paired *t* test; *p* < 0.021), and both CP volume at year two and CP enlargement correlated with sNfL levels (B = 231.9; SE = 52.3; *p* < 0.001; Figure 3A; Table S3; and B = 283.6; SE = 71.3; *p* < 0.001; Figure 3B; Table S4). Mean sNfL concentrations significantly decreased from baseline to follow-up (paired *t* test, *p* < 0.01), consistent with the initiation of DMT in the majority of patients. Despite this decline, the correlation between CP volume and sNfL levels remained comparable in strength to that observed at baseline (B = 309.8; SE = 112.8; *p* = 0.008; Figure 3C; Table S5).

### CP volume correlates with structural neurodegeneration markers

To further validate our findings, we examined the relationship between CP volume and cortical thickness as an imaging-based, structural marker of neurodegeneration. In the main cohort, higher CP volume was significantly associated with lower cortical thickness (B = −1,627.3; SE = 167.4; *p* < 0.001; Figure S1A). In the subcohort, CP enlargement over 2 years was associated with cortical atrophy (B = −6,075.6; SE = 2,804.6; *p* = 0.031; Figure S1B). Similarly, higher CP volume correlated with lower thalamic and subcortical GM volumes, and longitudinal CP enlargement was associated with subcortical atrophy (Figures S1C–S1F).

### CP volume is increased in secondary progressive MS

To ultimately validate our findings in the context of neurodegeneration, we examined CP volume across disease stages, comparing individuals with relapsing-remitting MS to a cohort of people with secondary progressive MS (*n* = 90), the latter representing a more neurodegeneration-driven phenotype. CP volume was significantly larger in secondary progressive than relapsing-remitting MS (*p* < 0.0001), and this difference persisted after adjustment for age (*p* = 0.023; Figures S2A and S2B).

## Discussion

This longitudinal multicenter study investigated the significance of CP size and enlargement for neuroaxonal injury in MS. Accordingly, we examined the association of the imaging-based marker CP volume and the blood-based biomarker sNfL. Our findings provide evidence for a strong and reproducible link between large CP volume and high sNfL levels in a large cohort of pwMS. High CP volume also predicted clinical progression as measured by EDSS worsening over a follow-up period of up to 6 years. Notably, after separating progression into its components, we detected that larger CP volume predicts PIRA but not RAW up to 6 years.

The positive correlation of high CP volume and importantly also CP enlargement after 2 years with the neuronal damage marker sNfL indicates that restructuring of the blood-CSF barrier accompanies loss of neuronal integrity, likely in a persistent rather than transient manner. Hence, the CP, traditionally recognized for its role in CSF production and for relaying inflammatory signals to the brain, may also play a pivotal role in the underlying neurodegenerative processes of MS, and it has been linked to disease severity.^8^ In fact, we here demonstrate a close temporal relationship between CP volume and sNfL levels as well as a correlation of sNfL levels with further CP enlargement over 2 years. This suggests that the CP may be more dynamically and earlier as well as sustainably involved in MS pathology than previously thought.

The relationship between CP enlargement and cortical atrophy provides cross-modal validation of our sNfL-based findings. The inverse association between CP volume and cortical thickness indicates that the CP may contribute to progressive tissue damage beyond acute inflammation in MS. As a real-time, dynamic biomarker, sNfL levels reflect ongoing neuroaxonal damage, capturing processes that may precede measurable structural atrophy.^9^ Cortical thinning rather depicts the cumulative result of past injury and may lag behind active pathological processes.

Neuropathological and imaging studies suggest that CP enlargement reflects sustained innate immune activation at the blood-CSF barrier.^4^^,^^10^ Activated macrophages and microglia at the blood-CSF barrier release cytokines and reactive oxygen species that diffuse through the CSF, fostering meningeal inflammation and cortical demyelination. Such chronic inflammatory signaling may underlie cortical atrophy and relapse-independent progression observed in our cohort.

The question arises whether neuronal injury depends on the function of the CP orchestrating immune cell infiltration of the blood-CSF barrier. What is apparent is that several CP pathological hallmarks, e.g., volume enlargement, appear conserved across different neurological diseases affecting the CNS,^11^ with immune cell infiltration within the CP being increased in autoimmunity.^12^

While CP enlargement in MS is viewed as a marker of pathological immune activation, it may also reflect adaptive or compensatory processes. Given the CP’s essential role in CNS homeostasis—including CSF production, metabolic clearance, immune regulation, and repair signaling—its enlargement might arise in response to sustained tissue stress or reduced CSF reduction, rather than representing purely deleterious inflammation.^13^ This multifaceted role highlights the need for mechanistic studies to determine whether CP enlargement in MS is a consequence of active immune cell infiltration or a secondary, adaptive response to systemic dysfunction.

Our findings point toward the prediction of PIRA by a CP volume over 1,526 mm^3^ (using FreeSurfer for CP segmentation)—a volume that exceeds the CP size of healthy subjects.^5^ In addition, our data indicate that CP volume predicts PIRA independently of sNfL levels. PIRA refers to the amount of accumulated neurological disability occurring independent of relapse activity, a feature that is believed to be linked to non-inflammatory neurodegeneration or compartmentalized inflammation. However, CP enlargement alone does not fully account for the development of PIRA, which likely arises from the interplay of multiple MRI-based markers reflecting both inflammatory and neurodegenerative processes.^14^ Based on current evidence, it is difficult to justify a disconnection between inflammatory pathology and neurodegeneration, but the data from our early MS cohort (disease duration less than 5 years) lead us to postulate that the enlarged CP is involved early in mechanisms leading to neuronal loss, demonstrated by high sNfL levels and concomitant relapse-free progression. Although specific pathophysiological mechanisms related to our observed association have not yet been solved, our findings highlight that CP enlargement manifests alongside neurodegenerative processes in MS. Notably, prior studies have reported associations between CP enlargement and both clinical and subclinical inflammatory activity,^5^^,^^10^^,^^15^ including relapses and MRI lesion burden; therefore, we cannot rule out that CP volume may correlate with RAW in other cohorts with higher overt inflammatory activity.

The role of DMTs in modulating CP volume and sNfL levels warrants consideration. High-efficacy DMTs are known to lower sNfL levels.^16^ In our regression models, DMT was a significant covariate, yet CP volume remained independently associated with sNfL levels, supporting a distinct contribution. Although the effect of DMTs on CP morphology remains incompletely understood, evidence suggests that high-efficacy treatment, in particular natalizumab, may modulate immune cell trafficking at the blood-CSF barrier and thereby mitigate CP enlargement.^5^

In this context, potential subtle effects of corticosteroids on CP volume and sNfL levels cannot be entirely excluded in our study, even though we adjusted for clinical relapses (which were largely treated with corticosteroids).

In clinical practice, CP enlargement could support early risk stratification, particularly in identifying pwMS at risk for progression despite the absence of clinical relapses. While its implementation requires further validation and standardization, our findings suggest that CP volume could complement existing imaging and fluid biomarkers in refining individualized treatment decisions.

In conclusion, we provide evidence for the utility of CP imaging as a complementary non-invasive biomarker for neuroaxonal loss already early in the disease and for the development of silent progression over time. CP enlargement predicts neuroaxonal loss in MS, suggesting that structural alterations of the blood-CSF barrier are linked to loss of neuronal integrity and, furthermore, disability worsening independent of acute exacerbations.

### Limitations of the study

Our study has some limitations. In humans, we cannot determine whether the relationship between CP enlargement and neuroaxonal loss is causatively linked. CP enlargement in MS is thought to be the result of immune cell aggregates in the CP stroma and vessels, with higher amounts of T lymphocytes, macrophages, and dendritic cells, and changes in the function and structure of ependymal cells including increased permeability of capillaries, thickening of the basement membrane, and loss of cilia in ependymal cells.^4^^,^^12^^,^^17^ Elevated CP volume may reflect structural and functional abnormalities of this physiological barrier associated with an inflammatory state. However, the underlying processes may not only promote acute inflammatory pathology but also neurodegenerative pathology, possibly via smoldering inflammation, in chronic MS.

Another limitation of our study may be the absence of routine spinal cord MRI. While symptomatic spinal relapses were captured and classified as RAW, approximately 12%–15% of spinal cord lesions may be asymptomatic and thus undetected.^18^ In addition, EDSS worsening in our study was not formally confirmed after 3 or 6 months as typically performed in clinical trials. However, all instances of EDSS progression (classified as PIRA or RAW) were sustained until the end of follow-up, supporting the robustness of the applied definition and reflecting real-world disease evolution within a large, longitudinal cohort.

Finally, while we applied a standardized imaging protocol across all centers and adjusted for scanner effects by including scanner as a covariate in all models, we did not apply advanced harmonization techniques such as ComBat.^19^ Although covariate adjustment is an accepted and effective method when acquisition protocols are uniform (as is the case for our study),^20^ residual site effects cannot be entirely excluded.

## Resource availability

### Lead contact

Further information and requests should be directed to and will be fulfilled by the lead contact, Vinzenz Fleischer (vinzenz.fleischer@unimedizin-mainz.de).

### Materials availability

This study did not generate new, unique reagents.

### Data and code availability

• Restrictions apply to the availability of these data and are therefore not publicly available. The raw data used in preparation of the figures and tables will be shared in anonymized format upon reasonable request by a qualified investigator for purposes of replicating procedures and results.
• This paper does not report original code.
• Any additional information required to reanalyze the data reported in this paper is available from the lead contact upon request.

## Acknowledgments

The authors thank all study participants as well as Dr. Cheryl Ernest for proofreading and editing the manuscript. The illustrations were adapted from BioRender.com templates (2021). This study was supported by the 10.13039/501100001659Deutsche Forschungsgemeinschaft (DFG; SFB CRC-TR-128 [project number 213904703] to F.Z., V.F., and S.B.; SFB 1080 [project number 221828878]; SFB CRC-1292 [project number 318346496] to F.Z.; and SFB/TRR 355 [project number 490846870] to S.B.), the 10.13039/100000890National Multiple Sclerosis Society (NMSS; grant no. RFA-2203-39314, subaward no. GMO 23101, and PO 0000002850 to V.F.), and the 10.13039/100017694Hermann and Lilly Schilling Foundation (to S.B.). The German Competence Network Multiple Sclerosis (KKNMS) was supported by grants from the German Federal Ministry for Education and Research (BMBF).

## Author contributions

V.F. and M.S. contributed to data analysis, writing, and figure design. V.F., M.S., G.G.-E., F.S., and T.B. performed data analysis and interpretation. A.S., T.B., F.S., R.S., M.P., N.H., S.G.M., F.L., H.W., L.K., R.G., C.L., S.B., S.G., and F.Z. edited the manuscript for important intellectual content. F.Z. initiated the study together with V.F. All authors had unrestricted access to all data. All authors agreed to submit the manuscript, read and approved the final draft, and take full responsibility for its content.

## Declaration of interests

V.F. has received research support from Novartis. R.S. has received speaker’s honoraria from Bayer HealthCare, Alexion Pharma, Novartis Pharma, and Roche Pharma AG; congress travel support from Merck and Biogen Idec GmbH; and research scientific grant support from Novartis Pharma. A.S. has received speaker honoraria from Bristol Myers Squibb, CSL Behring, Merck, Neuraxpharm, and Novartis and research support by the Baasch-Medicus Foundation, the Medical Faculty of the University of Bern, the Swiss MS Society, and the regional association of North Rhine-Westphalia of the German MS Society (DMSG Landesverband NRW), all not related to this work. S.G.M. has received honoraria for lecturing and travel expenses for attending meetings from Almirall, Amicus Therapeutics Germany, Bayer Health Care, Biogen, Bristol Myers Squibb/Celgene, Diamed, Genzyme, MedDay Pharmaceuticals, Merck Serono, Novartis, Novo Nordisk, Ono Pharma, Roche, Sanofi-Aventis, Chugai Pharma, QuintilesIMS, and Teva. His research is funded by the German Ministry for Education and Research, Bundesinstitut für Risikobewertung, Deutsche Forschungsgemeinschaft, Else Kröner Fresenius Foundation, Gemeinsamer Bundesausschuss, German Academic Exchange Service, Hertie Foundation, Interdisciplinary Center for Clinical Research Muenster, German Foundation Neurology, Alexion, Almirall, Amicus Therapeutics Germany, Biogen, Diamed, Fresenius Medical Care, Genzyme, Herz Burgdorf, Merck Serono, Novartis, Ono Pharma, Roche, and Teva. F.L. has received consultancy fees from Roche and support with travel cost from Teva Pharma. H.W. receives honoraria for acting as a member of Scientific Advisory Boards from Alexion, Argenx, BioCryst, Bristol Meyer Squibb/Celgene, Celerys, Galapagos, Janssen, Merck, Novartis, and Sandoz and speaker honoraria and travel support from Alexion, Biogen, Bristol Meyer Squibb, Genzyme, Merck, Neurodiem, Novartis, Ology, Roche, Teva, and WebMD Global. H.W. is a paid consultant for Actelion, Argenx, Beckton Dickinson, Bristol Meyer Squibb, Dianthus, EMD Serono, Fondazione Cariplo, Gossamer Bio, Idorsia, Immunic, Immunovant, INmune Bio_Syneos Health, Janssen, Lundbeck, LTS, Merck, NexGen, Novartis, Roche, Samsung, Sangamo, Sanofi-Aventis, the Swiss Multiple Sclerosis Society, Toleranzia, UCB Pharma GmbH, Viatris, VirBio, and Worldwide Clinical Trials; his research is funded by DFG, Deutsche Myasthenie Gesellschaft e.V., the European Union, Alexion, Amicus Therapeutics Inc., Argenx, Biogen, CSL Behring, F. Hoffmann-La Roche, Genzyme, Merck, Novartis, Roche, and UCB Pharma GmbH. L.K. has received compensation for serving on Scientific Advisory Boards for Alexion, Biogen, Bristol Myers Squibb, Hexal, Horizon, Janssen, Merck Serono, Novartis, Roche, Sandoz, Sanofi, Teva, and Viatris. She has received speaker honoraria and travel support from Argenx, Bayer, Biogen, Bristol Myers Squibb, Grifols, Horizon, Merck Serono, Novartis, Roche, Sanofi, Santhera, and Teva. She receives research support from the German Research Foundation, the IZKF Münster, Biogen, Novartis, and Merck Serono. R.G. has received compensation for serving as a consultant or speaker from Bayer HealthCare, Biogen Idec, Merck Serono, Novartis, and Teva Neuroscience; he, or the institution he works for, has received research support from Bayer HealthCare, Biogen Idec, Merck Serono, Novartis, and Teva Neuroscience; and he has also received honoraria as a Journal Editor from SAGE and Thieme Verlag. C.L. has received a research grant from the German Federal Ministry for Education and Research (BMBF), German Competence Network Multiple Sclerosis (KKNMS), grant no. 01GI 1601I, and has received consulting and speaker’s honoraria from Biogen Idec, Bayer Schering, Daiichi Sankyo, Merck Serono, Novartis, Sanofi, Genzyme, and Teva. S.B. has received honoraria and compensation for travel from Biogen Idec, Merck Serono, Novartis, Sanofi-Genzyme, and Roche. F.Z. has recently received research grants and/or consultation funds from DFG, BMBF, PMSA, MPG, Genzyme, Merck Serono, Roche, Novartis, Sanofi-Aventis, Celgene, ONO, and Octapharma.

## STAR★Methods

### Key resources table

**Table:** 

REAGENT or RESOURCE	SOURCE	IDENTIFIER
**Software and algorithms**		

GraphPad Prism 9.5.1	Graphstats Technologies	https://www.graphpad.com/
BioRender	BioRender	https://www.biorender.com/
FreeSurfer	FreeSurfer	https://surfer.nmr.mgh.harvard.edu/
IBM SPSS Statistics 26	IBM Corporation	https://www.ibm.com/products/spss

### Experimental model and study participant details

Within this prospective, longitudinal multicenter, pwMS were included over a period of 6 years. All were relapse-free and had received no corticosteroids for at least 30 days prior to enrollment. The study was performed in compliance with the Declaration of Helsinki and was approved by the local ethics committee. All pwMS provided written informed consent. Patients underwent clinical, laboratory and MRI evaluation at baseline. At each follow-up (year 1, 2, 4 and 6), patients were clinically reassessed and EDSS worsening and clinical relapses between visits were collected in order to define PIRA and RAW.^21^^,^^22^ In this study, PIRA was defined as an increase in EDSS score ≥1.0 point from baseline EDSS, occurring in the absence of a clinical relapse between the follow-up visits. RAW was defined as the same EDSS increase occurring in the presence of a documented clinical relapse between the follow-up visits. In this study, only sustained EDSS worsening was considered, meaning that all instances of EDSS increase (whether classified as PIRA or RAW) persisted until the end of the observational period. This approach ensured that transient fluctuations were excluded from progression definitions. A subcohort underwent a subsequent MRI examination at two-year follow-up.

Finally, an additional cohort of participants with secondary progressive MS was identified to examine CP volume across disease stages, given that this phenotype is clinically characterized by a more pronounced neurodegenerative trajectory. This cohort was compared with the individuals with relapsing-remitting MS to investigate CP differences linked to neurodegeneration-driven disability progression (Figure S2).

### Method details

#### MRI data acquisition

Conventional MRI images were acquired at different 3T scanners with a 32-channel receive-only head coil, according to a standardized imaging protocol in all centers. This protocol included sagittal 3D T1-weighted magnetization-prepared rapid gradient echo (MP-RAGE) and T2-weighted fluid-attenuated inversion recovery (FLAIR) sequences. All structural MRI datasets were collected and processed in one analyzing center (Mainz). A detailed description of all acquisition parameters is given in the supplement (Table S1).

#### Choroid plexus volume assessment

Semi-automated parcellation of CP in the lateral ventricles was performed from T1-weighted images using the open-source FreeSurfer software (Version 6.0),^23^^,^^24^ followed by visual inspection for quality control and adjustment as appropriate for each subject by an experienced scientist (GGE). FreeSurfer allows segmentation of the cortical and subcortical structures, including the thalamus and CP (Figure S1). To ensure that the results were not driven by other confounding factors, we also computed the intracranial volume and the lateral ventricle volume, and used them as additional independent covariates in the statistical models. Moreover, T2-hyperintense lesion volume and the presence of gadolinium–enhancing lesions were determined and included as covariates in the regression models. Technical details of the volume-based cortical and subcortical parcellation pipeline are described in the supplement.

#### sNfL measurements

All serum samples were processed according to the standardized biobanking protocol, which ensures harmonized procedures across all participating centers. Serum samples were collected by attending physicians, processed at room temperature within 2 hours, spun at 2000xg for 10 minutes, aliquoted using low-protein-binding polypropylene tubes and low-binding pipette tips to minimize adsorption effects. All aliquots were stored at −80°C until analysis. sNfL concentrations were measured in a blinded fashion as previously described using the highly sensitive single molecule array (SiMoA) technology.^25^^,^^26^

### Quantification and statistical analysis

To evaluate the impact of independent variables on CP volume, multiple regression models were created and adjusted for covariates (see supplemental information). MRI-based covariates were intracranial volume, lateral ventricle volume, T2-hyperintense lesion volume and the presence of gadolinium-enhancing lesions. Scanner/site (i.e., acquisition center) was included as a further categorical covariate in all regression models to account for potential inter-site variability. In a subset of participants with available data, body mass index (BMI) was included as an additional covariate in secondary regression analyses to assess its potential confounding effect. sNfL levels were log-transformed (log10) to ensure linearity. Kaplan-Meier method with Mantel-Cox and Mantel-Haenszel testing was applied to compare time to disability worsening (EDSS worsening, PIRA and RAW) between pwMS with low and high CP volume (excluding those patients with high-efficacy treatment at blood draw). To dichotomize CP volume for prognostication of EDSS worsening, PIRA and RAW receiver operating characteristics (ROC) analyses were performed to identify optimal cut-off values (based on the Youden index). To finally evaluate whether CP volume independently contributes to the risk of disability progression beyond the effects of sNfL levels, a multivariate Cox proportional hazards regression model was performed with CP volume and sNfL levels as covariates. *p* values < 0.05 were considered significant.

## Supplemental information

Document S1. Figures S1 and S2, Tables S1–S5, and Methods S1

Document S2. Article plus supplemental information

## References

[1] Jakimovski D., Bittner S., Zivadinov R., Morrow S.A., Benedict R.H., Zipp F., Weinstock-Guttman B. (2024). Multiple sclerosis. Lancet.

[2] Ghersi-Egea J.F., Strazielle N., Catala M., Silva-Vargas V., Doetsch F., Engelhardt B. (2018). Molecular anatomy and functions of the choroidal blood-cerebrospinal fluid barrier in health and disease. Acta Neuropathol..

[3] Tan L.Y., Cunliffe G., Hogan M.P., Yeo X.Y., Oh C., Jin B., Kang J., Park J., Kwon M.S., Kim M., Jung S. (2024). Emergence of the brain-border immune niches and their contribution to the development of neurodegenerative diseases. Front. Immunol..

[4] Vercellino M., Votta B., Condello C., Piacentino C., Romagnolo A., Merola A., Capello E., Mancardi G.L., Mutani R., Giordana M.T., Cavalla P. (2008). Involvement of the choroid plexus in multiple sclerosis autoimmune inflammation: a neuropathological study. J. Neuroimmunol..

[5] Fleischer V., Gonzalez-Escamilla G., Ciolac D., Albrecht P., Küry P., Gruchot J., Dietrich M., Hecker C., Müntefering T., Bock S. (2021). Translational value of choroid plexus imaging for tracking neuroinflammation in mice and humans. Proc. Natl. Acad. Sci. USA.

[6] Klistorner S., Van der Walt A., Barnett M.H., Butzkueven H., Kolbe S., Parratt J., Yiannikas C., Klistorner A. (2023). Choroid plexus volume is enlarged in clinically isolated syndrome patients with optic neuritis. Mult. Scler..

[7] Klistorner S., Barnett M.H., Parratt J., Yiannikas C., Graham S.L., Klistorner A. (2022). Choroid plexus volume in multiple sclerosis predicts expansion of chronic lesions and brain atrophy. Ann. Clin. Transl. Neurol..

[8] Bergsland N., Dwyer M.G., Jakimovski D., Tavazzi E., Benedict R.H.B., Weinstock-Guttman B., Zivadinov R. (2023). Association of choroid plexus inflammation on MRI with clinical disability progression over 5 years in patients with multiple sclerosis. Neurology.

[9] Barro C., Benkert P., Disanto G., Tsagkas C., Amann M., Naegelin Y., Leppert D., Gobbi C., Granziera C., Yaldizli Ö. (2018). Serum neurofilament as a predictor of disease worsening and brain and spinal cord atrophy in multiple sclerosis. Brain.

[10] Ricigliano V.A.G., Louapre C., Poirion E., Colombi A., Yazdan Panah A., Lazzarotto A., Morena E., Martin E., Bottlaender M., Bodini B. (2022). Imaging characteristics of choroid plexuses in presymptomatic multiple sclerosis: a retrospective study. Neurol. Neuroimmunol. Neuroinflamm..

[11] Tsitsou-Kampeli A., Suzzi S., Schwartz M. (2024). The immune and metabolic milieu of the choroid plexus as a potential target in brain protection. Trends Neurosci..

[12] Rodríguez-Lorenzo S., Konings J., Van Der Pol S., Kamermans A., Amor S., Van Horssen J., Witte M.E., Kooij G., De Vries H.E. (2020). Inflammation of the choroid plexus in progressive multiple sclerosis: accumulation of granulocytes and T cells. Acta Neuropathol. Commun..

[13] Alisch J.S.R., Kiely M., Triebswetter C., Alsameen M.H., Gong Z., Khattar N., Egan J.M., Bouhrara M. (2021). Characterization of age-related differences in the human choroid plexus volume, microstructural integrity, and blood perfusion using multiparameter magnetic resonance imaging. Front. Aging Neurosci..

[14] Bsteh G., Dal-Bianco A., Krajnc N., Berger T. (2025). Biomarkers of progression independent of relapse activity-can we actually measure it yet?. Int. J. Mol. Sci..

[15] Ricigliano V.A.G., Morena E., Colombi A., Tonietto M., Hamzaoui M., Poirion E., Bottlaender M., Gervais P., Louapre C., Bodini B., Stankoff B. (2021). Choroid plexus enlargement in inflammatory multiple sclerosis: 3.0-T MRI and translocator protein PET evaluation. Radiology.

[16] Novakova L., Zetterberg H., Sundström P., Axelsson M., Khademi M., Gunnarsson M., Malmeström C., Svenningsson A., Olsson T., Piehl F. (2017). Monitoring disease activity in multiple sclerosis using serum neurofilament light protein. Neurology.

[17] Preziosa P., Pagani E., Meani A., Storelli L., Margoni M., Yudin Y., Tedone N., Biondi D., Rubin M., Rocca M.A., Filippi M. (2024). Chronic active lesions and larger choroid plexus explain cognition and fatigue in multiple sclerosis. Neurol. Neuroimmunol. Neuroinflamm..

[18] Ostini C., Bovis F., Disanto G., Ripellino P., Pravatà E., Sacco R., Padlina G., Sormani M.P., Gobbi C., Zecca C. (2021). Recurrence and prognostic value of asymptomatic spinal cord lesions in multiple sclerosis. J. Clin. Med..

[19] Fortin J.P., Cullen N., Sheline Y.I., Taylor W.D., Aselcioglu I., Cook P.A., Adams P., Cooper C., Fava M., McGrath P.J. (2018). Harmonization of cortical thickness measurements across scanners and sites. Neuroimage.

[20] Pomponio R., Erus G., Habes M., Doshi J., Srinivasan D., Mamourian E., Bashyam V., Nasrallah I.M., Satterthwaite T.D., Fan Y. (2020). Harmonization of large MRI datasets for the analysis of brain imaging patterns throughout the lifespan. Neuroimage.

[21] Lublin F.D., Häring D.A., Ganjgahi H., Ocampo A., Hatami F., Čuklina J., Aarden P., Dahlke F., Arnold D.L., Wiendl H. (2022). How patients with multiple sclerosis acquire disability. Brain.

[22] Müller J., Cagol A., Lorscheider J., Tsagkas C., Benkert P., Yaldizli Ö., Kuhle J., Derfuss T., Sormani M.P., Thompson A., Granziera C. (2023). Harmonizing definitions for progression independent of relapse activity in multiple sclerosis: a systematic review. JAMA Neurol..

[23] Desikan R.S., Ségonne F., Fischl B., Quinn B.T., Dickerson B.C., Blacker D., Buckner R.L., Dale A.M., Maguire R.P., Hyman B.T. (2006). An automated labeling system for subdividing the human cerebral cortex on MRI scans into gyral based regions of interest. Neuroimage.

[24] Fischl B. (2012). FreeSurfer. Neuroimage.

[25] Bittner S., Steffen F., Uphaus T., Muthuraman M., Fleischer V., Salmen A., Luessi F., Berthele A., Klotz L., Meuth S.G. (2020). Clinical implications of serum neurofilament in newly diagnosed MS patients: A longitudinal multicentre cohort study. EBioMedicine.

[26] Schuckmann A., Steffen F., Zipp F., Bittner S., Pape K. (2023). Impact of extended interval dosing of ocrelizumab on immunoglobulin levels in multiple sclerosis. Med.

