# Supplementary material for: Choroid plexus enlargement associates with serum neurofilament and predicts relapse-free progression in multiple sclerosis
Source: Cell Rep Med. 2026 Feb 9;7(2):102609. doi: 10.1016/j.xcrm.2026.102609 (PMC12923943; doi:10.1016/j.xcrm.2026.102609)
Supplement: Document S1. Figures S1 and S2, Tables S1–S5, and Methods S1 [file mmc1.pdf]

Cell Reports Medicine, Volume *7*

Supplemental information

Choroid plexus enlargement associates with serum neurofilament and predicts relapse-free progression in multiple sclerosis

Vinzenz Fleischer, Muriel Schraad, Gabriel Gonzalez-Escamilla, Ruth Schneider, Tobias Brummer, Falk Steffen, Maria Protopapa, Nicholas Hanuscheck, Anke Salmen, Sven G. Meuth, Felix Luessi, Heinz Wiendl, Luisa Klotz, Ralf Gold, Carsten Lukas, Stefan Bittner, Sergiu Groppa, and Frauke Zipp

SUPPLEMENTAL INFORMATION

Methods S1: MRI preprocessing pipeline and FreeSurfer-based parcellation procedures for CP volume quantification

Detailed acquisition parameters are found in Supplementary Table 1. Before pre-processing, the nonparametric non-uniform normalization correction was applied to all images to reduce intensity non-uniformity. For all participants, semi-automated parcellation of the choroid plexus (CP) in the lateral ventricles was performed from T1-weighted images using FreeSurfer (version 6.0; https://surfer.nmr.mgh.harvard.edu/). To ensure the robustness of CP volume measurements, a subset of scans was independently re-segmented by two raters, and visual inspection confirmed a high degree of consistency between segmentations, with no systematic deviations observed. Briefly, a probabilistic atlas, built by manual labeling on a training dataset normalized to the MNI305 space resulting in a point-to-point correspondence between all training subjects, is used as parcellation prior for all brain regions [1,2]. This atlas provides the probability of each brain region to belong to a given voxel, the probability of each brain region given the classification of neighboring voxels (neighborhood function), and the probability distribution function of voxel intensities, modelled as a normal distribution, for each brain region at each voxel. Then, newly introduced images are parcelled by normalizing the new image to the common space and incorporating the subject-specific voxel intensities to find the optimal parcellation that maximizes the probability of observing the input data. To ensure that the results including CP volumes are not driven by other confounding factors, we also extracted total intracranial volume and lateral ventricle volume. Following brain CP and ventricle parcellation by tiling the boundary of white matter mass, an initial white surface is created for each cerebral hemisphere, which is further refined following intensity gradients of the white and gray matter to generate the final gray–white surface. This surface is then extended to follow the intensity gradient of gray matter and cerebrospinal fluid (CSF), leading to the creation of the pial surface. Finally, cortical thickness at each surface vertex is computed as the average distance from each vertex in the gray–white surface to the corresponding point in the pial surface [1,2].

Supplementary Figure S1. Association of CP volume and MRI-derived atrophy measures. Related to STAR Methods and the Results. (A) Association between CP volume and cortical thickness in pwMS (sex- and age-adjusted). **(B)** Association between CP volume change and cortical thickness change over two years (sex- and age-adjusted). **(C)** Association between CP volume and thalamic volume (sex- and age-adjusted). **(D)** Association between CP volume change and thalamic volume change (atrophy) over two years (sex- and age-adjusted). **(E)** Association between CP volume and subcortical grey matter (GM) volume (sex- and age-adjusted). **(F)** Association between CP volume change and subcortical GM volume change (atrophy) over two years (sex- and age-adjusted). Linear fits (red solid lines) to individual data points are shown with 95% confidence intervals (dotted lines).

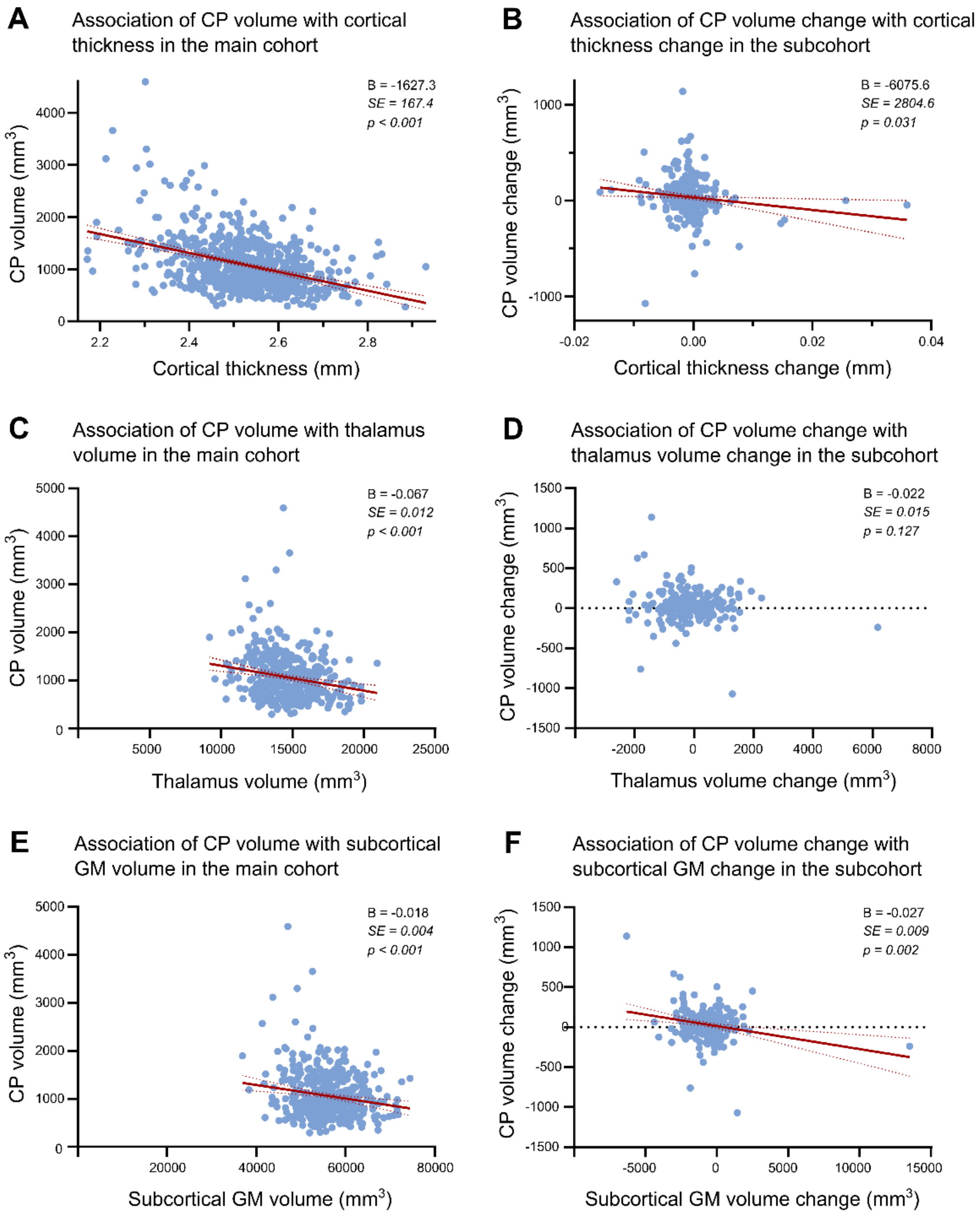

Supplementary Figure S2. CP enlargement in relapsing-remitting MS (RRMS) compared to a cohort with secondary progressive MS (SPMS). Related to STAR Methods and the Results. (A) Comparison of absolute CP volume between people with RRMS and SPMS (n=90), showing significantly higher CP volumes in the SPMS group. **(B)** Comparison of CP volume normalized to age (CP volume/age) between RRMS and SPMS, demonstrating that the difference remains significant after age correction. Violin plots depict the full data distribution with median values indicated by horizontal lines.

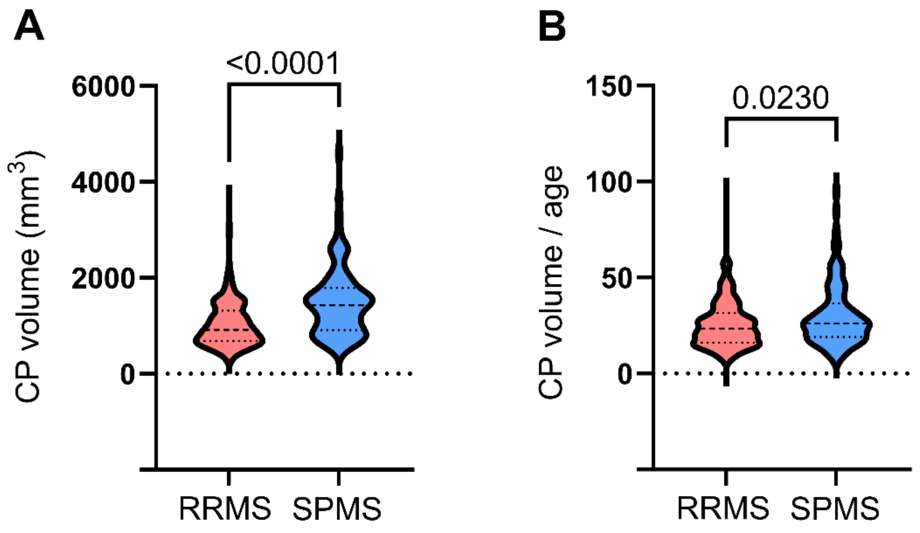

Supplementary Table 1. MR scanners and image acquisition parameters of the centers. Related to STAR Methods.

Center	Scanner (vendor)	No. of patients	3D T1-weighted sequence	3D T2-weighted FLAIR sequence
Mainz	Magnetom Trio (Siemens)	274	192 sagittal slices with 1 mm isotropic voxel size and a 256 x 256 matrix size TE/TI/TR = 2.52/900/1900 ms	192 sagittal slices with 1 mm isotropic voxel size and a 256 x 256 matrix size TE/TI/TR = 388/1800/5000 ms
Münster	Magnetom Prisma (Siemens)	87	192 sagittal slices with 1 mm isotropic voxel size and a 256 x 256 matrix size TE/TI/TR = 2.2/900/2130 ms	192 sagittal slices with 1 mm isotropic voxel size and a 256 x 256 matrix size TE/TI/TR = 389/1800/5000 ms
Bochum	Achieva (Philips)	71	180 sagittal slices with 1 mm isotropic voxel size and a 256 x 256 matrix size TE/TI/TR = 4.6/1000/10 ms	170 sagittal slices with 1 mm isotropic voxel size and 256 x 256 matrix size TE/TI/TR = 286/1650/4800 ms

Supplementary Table 2. Regression model on MRI-derived CP volume at baseline. Related to Figure 2A. Linear regression model of covariables influencing the association between CP volume and sNfL levels in pwMS who underwent baseline MRI (n=434). The regression model was corrected for sex, age, disease duration, Expanded Disability Status Scale (EDSS) score, disease-modifying treatment (DMT), intracranial volume, scanner and lateral ventricle volume. T2 lesion volume (n=326) was inserted into the regression model; the presence of gadolinium (Gad)-enhancing lesions (n=276) and body mass index (BMI; n=177) were subsequently inserted into that regression model. Regression coefficients B with 95% confidence interval (lower and upper bound) and level of significance are given. P values <0.05 were considered significant.

Independent variables	Regression coefficient B	p-value	Lower bound 95%CI	Upper bound 95%CI
**Sex**	-49.719	*0.119*	-112.213	12.775
**Age**	3.741	*0.003*	1.288	6.193
**Disease duration**	0.482	*0.284*	-0.402	1.366
**EDSS**	-5.000	*0.676*	-28.536	18.536
**DMT**	108.502	*0.073*	-10.291	227.296
**Intracranial volume**	0.000	*0.263*	0.000	0.000
**Scanner**	-38.742	*0.195*	-97.352	19.868
**Lateral ventricle volume**	0.033	*<0.001*	0.030	0.035
**sNfL (log_{10})**	125.769	*<0.001*	56.573	194.965
**T2 lesion volume**	4.929	*<0.001*	2.627	7.231
**Gad-enhancing lesions**	49.878	*0.173*	-22.039	121.796
**BMI**	-5.834	*0.093*	-12.648	0.979

Supplementary Table 3. Regression model on MRI-derived CP volume after two years. Related to Figure 3A. Linear regression model of covariables influencing the association between the change in CP volume and sNfL levels in pwMS who underwent baseline and 2-year follow-up MRI (n=208). The regression model was corrected for sex, age, disease duration, Expanded Disability Status Scale (EDSS) score, disease-modifying treatment (DMT), intracranial volume, lateral ventricle volume, number of relapses between the MRIs and time between the MRIs. T2 lesion volume at baseline (n=208) was inserted into the regression model; the presence of gadolinium (Gad)-enhancing lesions at baseline (n=208) and body mass index (BMI; n=70) were subsequently inserted into that regression model. Regression coefficients B with 95% confidence interval (lower and upper bound) and level of significance are given. P values <0.05 were considered significant.

Independent variables	Regression coefficient B	p-value	Lower bound 95%CI	Upper bound 95%CI
**Sex**	-183.815	*0.002*	-297.521	-70.109
**Age**	3.208	*0.101*	-0.627	7.043
**Disease duration**	-0.511	*0.323*	-1.526	0.505
**EDSS**	-4.509	*0.779*	-36.174	27.155
**DMT**	58.422	*0.292*	-50.555	167.400
**Intracranial volume**	-0.001	*0.021*	-0.003	-0.001
**Lateral ventricle volume**	0.038	*<0.001*	0.034	0.042
**No. of relapses between MRIs**	9.586	*0.676*	-35.527	54.700
**Time between MRIs**	0.920	*0.502*	-1.779	3.619
**sNfL (log_{10})**	231.904	*<0.001*	128.815	334.993
**T2 lesion volume**	7.485	*0.005*	2.338	12.632
**Gad-enhancing lesions**	60.739	*0.228*	-38.25	159.729
**BMI**	-5.019	*0.489*	-19.442	9.403

Supplementary Table 4. Regression model on MRI-derived CP volume change over two years. Related to Figure 3B. Linear regression model of covariables influencing the association between the change in CP volume in pwMS who underwent baseline and 2-year follow-up MRI and displayed CP enlargement (n=122). The regression model was corrected for sex, age, disease duration, Expanded Disability Status Scale (EDSS) change, disease-modifying treatment (DMT), intracranial volume, lateral ventricle volume, number of relapses between the MRIs and time between the MRIs. T2 lesion volume at baseline (n=122) was inserted into the regression model; the presence of gadolinium (Gad)-enhancing lesions at baseline (n=122) and body mass index (BMI; n=43) were subsequently inserted into that regression model. Regression coefficients B with 95% confidence interval (lower and upper bound) and level of significance are given. P values <0.05 were considered significant.

Independent variables	Regression coefficient B	p-value	Lower bound 95%CI	Upper bound 95%CI
**Sex**	-161.013	*0.072*	-336.854	14.829
**Age**	5.398	*0.069*	-0.431	11.226
**Disease duration**	-0.614	*0.503*	-2.421	1.194
**EDSS change**	-34.868	*0.151*	-82.645	12.909
**DMT**	67.711	*0.400*	-91.23	226.652
**Intracranial volume**	-0.001	*0.048*	-0.002	-0.001
**Lateral ventricle volume**	0.042	*<0.001*	0.035	0.048
**No. of relapses between MRIs**	53.98	*0.164*	-22.388	130.348
**Time between MRIs**	0.412	*0.841*	-3.639	4.463
**sNfL (log_{10})**	283.627	*<0.001*	142.322	424.932
**T2 lesion volume**	13.041	*0.003*	4.456	21.625
**Gad-enhancing lesions**	21.008	*0.754*	-111.467	153.482
**BMI**	-3.622	*0.727*	-3.622	-3.622

Supplementary Table 5. Regression model on MRI-derived CP volume after two years. Related to Figure 3C. Linear regression model of covariables influencing the association between the CP volume and sNfL levels in pwMS who underwent 2-year follow-up MRI and sNfL measurement (n=81). The regression model was corrected for sex, age, disease duration, Expanded Disability Status Scale (EDSS) score, disease-modifying treatment (DMT), intracranial volume, lateral ventricle volume, and T2 lesion volume at 2 years. Regression coefficients B with 95% confidence interval (lower and upper bound) and level of significance are given. P values <0.05 were considered significant.

Independent variables	Regression coefficient B	p-value	Lower bound 95%CI	Upper bound 95%CI
**Sex**	-235.816	*0.005*	-397.260	-74.372
**Age**	-0.891	*0.709*	-5.631	3.849
**Disease duration**	-5.496	*0.000*	-7.710	-3.282
**EDSS**	-28.656	*0.201*	-72.904	15.592
**DMT**	85.883	*0.192*	-44.043	215.810
**Intracranial volume**	-0.001	*0.048*	-0.002	-0.001
**Lateral ventricle volume**	0.042	*<0.001*	0.036	0.047
**sNfL (log_{10})**	309.798	*0.008*	84.871	534.726
**T2 lesion volume**	7.210	*0.008*	1.908	12.512

References

1. Fischl, B. (2012). FreeSurfer. Neuroimage *62*, 774-781. 10.1016/j.neuroimage.2012.01.021.
2. Desikan, R.S., Segonne, F., Fischl, B., Quinn, B.T., Dickerson, B.C., Blacker, D., Buckner, R.L., Dale, A.M., Maguire, R.P., Hyman, B.T., et al. (2006). An automated labeling system for subdividing the human cerebral cortex on MRI scans into gyral based regions of interest. Neuroimage *31*, 968-980. 10.1016/j.neuroimage.2006.01.021.
