# Supplementary material for: Choroid plexus enlargement associates with serum neurofilament and predicts relapse-free progression in multiple sclerosis
Source: Cell Rep Med. 2026 Feb 9;7(2):102609. doi: 10.1016/j.xcrm.2026.102609 (PMC12923943; doi:10.1016/j.xcrm.2026.102609)
Supplement: Document S2. Article plus supplemental information [file mmc2.pdf]

Cell Reports Medicine

Report

Choroid plexus enlargement associates with serum neurofilament and predicts relapse-free progression in multiple sclerosis

Graphical abstract

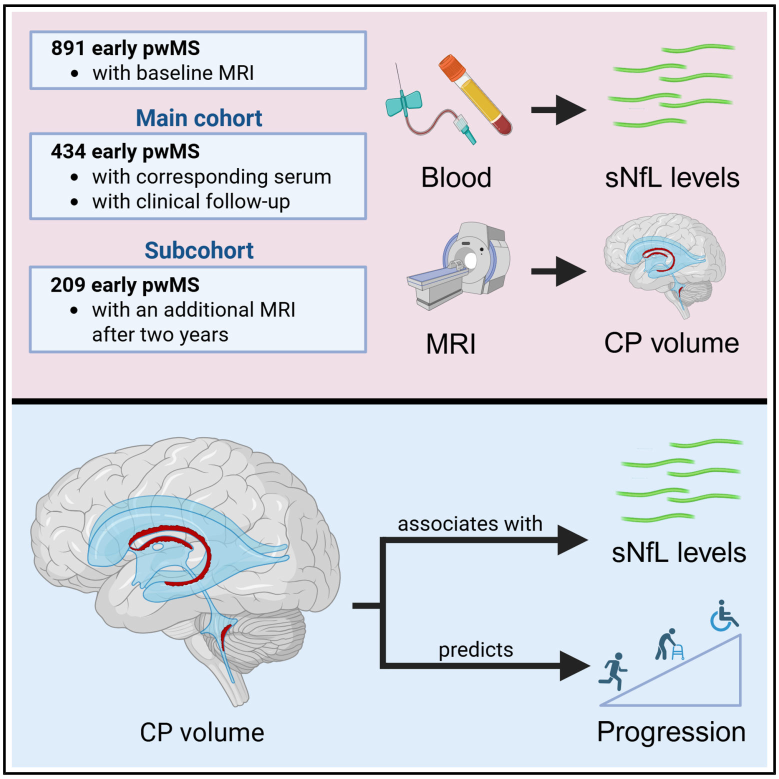

Authors

Vinzenz Fleischer, Muriel Schraad, Gabriel Gonzalez-Escamilla, ..., Stefan Bittner, Sergiu Groppa, Frauke Zipp

Correspondence

vinzenz.fleischer@unimedizin-mainz.de

In brief

In this multicenter longitudinal study, Fleischer et al. show that enlargement of the choroid plexus, a key gateway between the blood and cerebrospinal fluid, signals early neuroaxonal injury and predicts silent progression in MS. Their findings suggest that volumetric changes at this barrier contribute to neurodegeneration and clinical disability in MS.

Highlights

- Choroid plexus (CP) volume associates with serum neurofilament levels in MS
- CP enlargement over time reflects ongoing neuroaxonal injury
- High CP volume predicts relapse-free progression in people with MS

Fleischer et al., 2026, Cell Reports Medicine 7, 102609
February 17, 2026 © 2026 The Author(s). Published by Elsevier Inc.
https://doi.org/10.1016/j.xcrm.2026.102609

CellPress

Cell Reports Medicine

CellPress
OPEN ACCESS

Report

Choroid plexus enlargement associates with serum neurofilament and predicts relapse-free progression in multiple sclerosis

Vinzenz Fleischer,[1,7,8,*] Muriel Schraad,[1,7] Gabriel Gonzalez-Escamilla,[1] Ruth Schneider,[2] Tobias Brummer,[1] Falk Steffen,[1] Maria Protopapa,[1] Nicholas Hanuscheck,[1] Anke Salmen,[2] Sven G. Meuth,[3] Felix Luessi,[1] Heinz Wiendl,[4] Luisa Klotz,[5] Ralf Gold,[2] Carsten Lukas,[6] Stefan Bittner,[1] Sergiu Groppa,[1] and Frauke Zipp[1]

[1]Department of Neurology, Research Center for Immunotherapy (FZI) and Focus Program Translational Neuroscience (FTN), Rhine Main Neuroscience Network (rmn2), University Medical Center of the Johannes Gutenberg University Mainz, 55131 Mainz, Germany
[2]Department of Neurology, St. Josef-Hospital, Ruhr-University Bochum, 44791 Bochum, Germany
[3]Department of Neurology, University Hospital Düsseldorf, Medical Faculty, Heinrich Heine University, 40225 Düsseldorf, Germany
[4]Department of Neurology and Clinical Neuroscience, Faculty of Medicine, Medical Center University of Freiburg, 79106 Freiburg, Germany
[5]Department of Neurology, University Hospital Münster, Westfälische-Wilhelms-University Münster, 48149 Münster, Germany
[6]Institute of Neuroradiology, St. Josef Hospital, Ruhr University Bochum, 44791 Bochum, Germany
[7]These authors contributed equally
[8]Lead contact
*Correspondence: vinzenz.fleischer@unimedizin-mainz.de
https://doi.org/10.1016/j.xcrm.2026.102609

SUMMARY

The choroid plexus (CP), a key regulator of cerebrospinal fluid production and immune cell trafficking, is increasingly recognized as a potential magnetic resonance imaging (MRI) biomarker in multiple sclerosis (MS). Serum neurofilament light chain (sNfL) serves as a sensitive blood-based indicator of neuroaxonal injury. We investigate the prognostic value of CP volume and its longitudinal change for neurodegeneration, defined by sNfL levels and disability progression. In a prospective, multicenter, longitudinal study, 891 people with MS undergo high-resolution 3T MRI, sNfL measurement, and clinical assessment over 6 years; 434 meet criteria for inclusion. CP volume correlates with sNfL after adjustment for demographic, clinical, treatment, and imaging variables. In a 2-year MRI subcohort (n = 209), CP enlargement likewise associates with sNfL. High CP volume confers a 1.8-fold increased risk of disability worsening and a 2.7-fold increased risk of progression independent of relapse activity. These findings identify CP imaging as a promising non-invasive biomarker of neuroaxonal loss and relapse-free progression.

INTRODUCTION

Multiple sclerosis (MS) is characterized by neuroinflammation, demyelination, and neuroaxonal damage.[1] Neuroimaging studies have been instrumental in elucidating the pathological processes underlying MS, with magnetic resonance imaging (MRI) serving as a cornerstone for diagnosis and monitoring.

Compelling evidence supports an essential role of the choroid plexus (CP) as a key invasion route for immune cells into the CNS and thus as part of immune surveillance.[2] The CP separates the cerebrospinal fluid (CSF) from blood and serves as an immunological niche.[3] In MS, immune cells invading the ventricles have to eventually pass the CP epithelium.[2] Histopathologic findings demonstrated an enlargement of the CP in MS due to edema with high concentrations of T lymphocytes, dendritic cells, and activated macrophages.[4] On the macroscale, CP enlargement was confirmed with advanced imaging algorithms in high-resolution MRI in people with MS (pwMS).[5] Greater CP volume was associated with increased lesion activity and greater brain atrophy,[6,7] suggesting that CP enlargement promotes the inflammatory part of the pathology.

Therefore, we aimed to explore the association between CP enlargement and serum neurofilament light (sNfL) levels, as a marker of neuroaxonal loss, in a large longitudinal multicenter cohort of pwMS early in the disease course and to find out whether CP volume has prognostic value for future disability worsening. In this report, we found that increased CP volume associated with early neuroaxonal loss and predicted the development of silent disease progression over time, suggesting that the CP is very early, dynamically as well as sustainably involved in MS neuronal compartment pathology.

RESULTS

Study cohort characteristics

An overview of the demographics and clinical characteristics of the main cohort and subcohort is depicted in Table 1.

In total, 891 pwMS with standardized 3 Tesla MRI were screened; 434 patients fulfilled the imaging requirements for

© 2026 The Author(s). Published by Elsevier Inc.
This is an open access article under the CC BY license (http://creativecommons.org/licenses/by/4.0/).

Table 1. Basic data

	Main cohort (n = 434)	Subcohort (n = 209)
Sex (female/male)	310/124	149/60
Age at MRI, mean (±SD) (years)	38.7 ± 12.0	42.9 ± 12.4
Disease duration, mean (±SD) (years)	1.6 ± 3.2	2.6 ± 4.2
EDSS score, median (range) (at baseline)	1.5 (0–7.5)	1.0 (0–7.5)
EDSS score, median (range) (at follow-up)	–	1.5 (0–7.5)
DMT baseline (none/platform/high efficacy)	271/129/34	56/121/32
DMT follow-up (none/platform/high efficacy)	–	32/128/49
Duration of MRI follow-up, mean (±SD) (years)	–	2.4 ± 1.3
Serum NfL, mean (±SD) (pg/mL)	17.4 ± 27.3	17.0 ± 31.6
Serum NfL follow-up, mean (±SD) (pg/mL)	–	10.2 ± 15.7
CP, mean (±SD) (at baseline; mm^3)	1,066.3 ± 488.9	1,078.3 ± 573.3
CP, mean (±SD) (at follow-up; mm^3)	–	1,111.4 ± 569.5
Change of plexus volume, mean (±SD) (mm^3)	–	33.1 ± 197.6

Demographical and clinical data as well as MRI (CP volume) and blood-based (sNfL levels) measures of people with MS (pwMS) in the main cohort (n = 434) and the subcohort (n = 209). CP, choroid plexus; EDSS, Expanded Disability Status Scale; DMT, disease-modifying treatment; MRI, magnetic resonance imaging; SD, standard deviation; sNfL, serum neurofilament.

CP volume measurement, had serum available at the imaging time point, and were enrolled in the main study. Figure 1 outlines our study design. The mean (±standard deviation [SD]) age of the included 434 pwMS was 38.7 ± 12.0 years; 310 (71.4%) patients were female and 124 (28.6%) were male. Mean disease duration at MRI and serum acquisition was 1.6 ± 3.2 years; median disability (quantified by Expanded Disability Status Scale [EDSS]) was 1.0 (range 0–7.5). Thirty-four pwMS were on high-efficacy disease-modifying treatment (DMT; natalizumab, alemtuzumab, anti-CD20 antibodies, or sphingosine-1-phosphate receptor modulators).

In the subcohort with an additional MRI after 2 years (n = 209), the mean (±SD) age was 42.9 ± 12.4 years; 149 (71.3%) patients were female and 60 (28.7%) were male. Mean disease duration at baseline MRI was 2.6 ± 4.2 years; median disability (quantified by EDSS) was 1.0 (range 0–7.5). Thirty-two pwMS were on high-efficacy DMT.

Association of CP volume with sNfL levels and the predictive capacity of CP volume for disability accumulation in MS

Overall, the results from the main cohort demonstrated a correlation of CP volume with sNfL levels in pwMS in the regression model correcting for sex, age, disease duration, EDSS score, DMT, intracranial volume, scanner, lateral ventricle volume, T2 lesion volume, gadolinium-enhancing lesions, and BMI (Figure 2A) (B = 125.8; SE = 35.2; $p < 0.001$). Significant influential covariates in the main cohort were age, lateral ventricle volume, and T2 lesion volume (Table S2).

Kaplan-Meier analysis using dichotomized CP volume was applied to predict time to disability worsening. Dichotomized patient groups were based on the optimized cutoff values of CP volume. The associated criterion of the Youden index was a CP volume of 1,526 mm^3 (CP volume < 1,526 mm^3 = "CP volume^{low}" and CP volume > 1,526 mm^3 = "CP volume^{high}"). Thereby, pwMS with a CP volume^{high} revealed a 1.8-fold increased risk of EDSS worsening compared to CP volume^{low} at baseline (95% confidence interval [CI] = 1.087–2.812; p = 0.021; Figure 2B). We then applied Kaplan-Meier analysis to predict PIRA (progression independent of relapse activity) or RAW (relapse-associated worsening) versus patients who did not attain PIRA or RAW. PIRA was defined as an increase in EDSS score that occurred in the absence of a clinical relapse, while RAW was defined as an increase in EDSS score that occurred after a documented clinical relapse. CP volume^{high} revealed a 2.7-fold risk of PIRA compared to CP volume^{low} (95% CI = 1.240–5.649; p = 0.012; Figure 2C), while CP volume was not significantly different in the time-to-event analysis of RAW (p = 0.123; Figure 2D). In an additional multivariate Cox regression analysis adjusting for sNfL levels, CP volume remained a significant predictor of increased risk for PIRA (hazard ratio = 1.95; 95% CI = 1.05–3.62; p = 0.035).

Association of CP volume with sNfL levels in the subcohort with longitudinal MRI

In those pwMS with a follow-up MRI acquired using the same protocol and scanner as at baseline, mean CP volume increased over 2 years (paired t test; $p < 0.021$), and both CP volume at year two and CP enlargement correlated with sNfL levels (B = 231.9; SE = 52.3; $p < 0.001$; Figure 3A; Table S3; and B = 283.6; SE = 71.3; $p < 0.001$; Figure 3B; Table S4). Mean sNfL concentrations significantly decreased from baseline to follow-up (paired t test, $p < 0.01$), consistent with the initiation of DMT in the majority of patients. Despite this decline, the correlation between CP volume and sNfL levels remained comparable in strength to that observed at baseline (B = 309.8; SE = 112.8; p = 0.008; Figure 3C; Table S5).

CP volume correlates with structural neurodegeneration markers

To further validate our findings, we examined the relationship between CP volume and cortical thickness as an imaging-based, structural marker of neurodegeneration. In the main cohort, higher CP volume was significantly associated with lower

CellPress
OPEN ACCESS

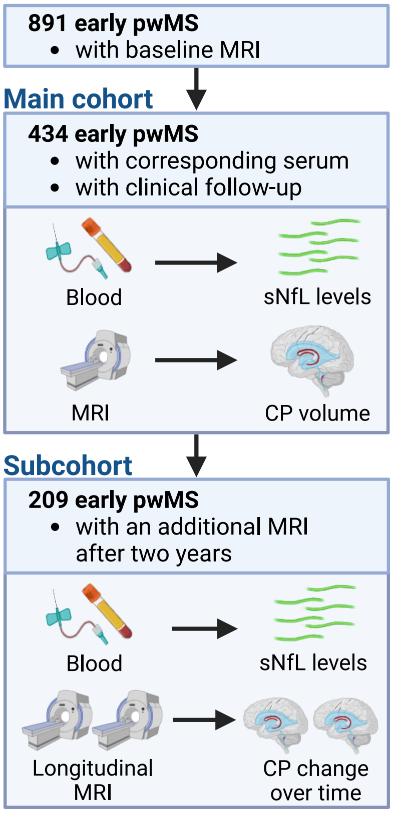

Figure 1. Study design

891 early pwMS (time since diagnosis <5 years) underwent baseline high-resolution MRI with standardized protocol in all centers. Out of these, 434 pwMS had serum available for sNfL measurement. In a subcohort of 209 pwMS, a subsequent MRI was performed after 2 years of follow-up.

cortical thickness ($B = -1,627.3$; $SE = 167.4$; $p < 0.001$; Figure S1A). In the subcohort, CP enlargement over 2 years was associated with cortical atrophy ($B = -6,075.6$; $SE = 2,804.6$; $p = 0.031$; Figure S1B). Similarly, higher CP volume correlated with lower thalamic and subcortical GM volumes, and longitudinal CP enlargement was associated with subcortical atrophy (Figures S1C–S1F).

CP volume is increased in secondary progressive MS

To ultimately validate our findings in the context of neurodegeneration, we examined CP volume across disease stages, comparing individuals with relapsing-remitting MS to a cohort of people with secondary progressive MS ($n = 90$), the latter representing a more neurodegeneration-driven phenotype. CP volume was significantly larger in secondary progressive than relapsing-remitting MS ($p < 0.0001$), and this difference persisted after adjustment for age ($p = 0.023$; Figures S2A and S2B).

DISCUSSION

This longitudinal multicenter study investigated the significance of CP size and enlargement for neuroaxonal injury in MS. Accordingly, we examined the association of the imaging-based marker CP volume and the blood-based biomarker sNfL. Our findings provide evidence for a strong and reproducible link between large CP volume and high sNfL levels in a large cohort of pwMS. High CP volume also predicted clinical progression as measured by EDSS worsening over a follow-up period of up to 6 years. Notably, after separating progression into its components, we detected that larger CP volume predicts PIRA but not RAW up to 6 years.

The positive correlation of high CP volume and importantly also CP enlargement after 2 years with the neuronal damage marker sNfL indicates that restructuring of the blood-CSF barrier accompanies loss of neuronal integrity, likely in a persistent rather than transient manner. Hence, the CP, traditionally recognized for its role in CSF production and for relaying inflammatory signals to the brain, may also play a pivotal role in the underlying neurodegenerative processes of MS, and it has been linked to disease severity.[8] In fact, we here demonstrate a close temporal relationship between CP volume and sNfL levels as well as a correlation of sNfL levels with further CP enlargement over 2 years. This suggests that the CP may be more dynamically and earlier as well as sustainably involved in MS pathology than previously thought.

The relationship between CP enlargement and cortical atrophy provides cross-modal validation of our sNfL-based findings. The inverse association between CP volume and cortical thickness indicates that the CP may contribute to progressive tissue damage beyond acute inflammation in MS. As a real-time, dynamic biomarker, sNfL levels reflect ongoing neuroaxonal damage, capturing processes that may precede measurable structural atrophy.[9] Cortical thinning rather depicts the cumulative result of past injury and may lag behind active pathological processes.

Neuropathological and imaging studies suggest that CP enlargement reflects sustained innate immune activation at the blood-CSF barrier.[4,10] Activated macrophages and microglia at the blood-CSF barrier release cytokines and reactive oxygen species that diffuse through the CSF, fostering meningeal inflammation and cortical demyelination. Such chronic inflammatory signaling may underlie cortical atrophy and relapse-independent progression observed in our cohort.

The question arises whether neuronal injury depends on the function of the CP orchestrating immune cell infiltration of the blood-CSF barrier. What is apparent is that several CP pathological hallmarks, e.g., volume enlargement, appear conserved across different neurological diseases affecting the CNS,[11] with immune cell infiltration within the CP being increased in autoimmunity.[12]

While CP enlargement in MS is viewed as a marker of pathological immune activation, it may also reflect adaptive or compensatory processes. Given the CP's essential role in CNS homeostasis—including CSF production, metabolic clearance, immune regulation, and repair signaling—its enlargement might arise in response to sustained tissue stress or reduced CSF reduction, rather than representing purely deleterious inflammation.[13] This multifaceted role highlights the need for mechanistic studies to determine whether CP enlargement in MS is a consequence of active immune cell infiltration or a secondary, adaptive response to systemic dysfunction.

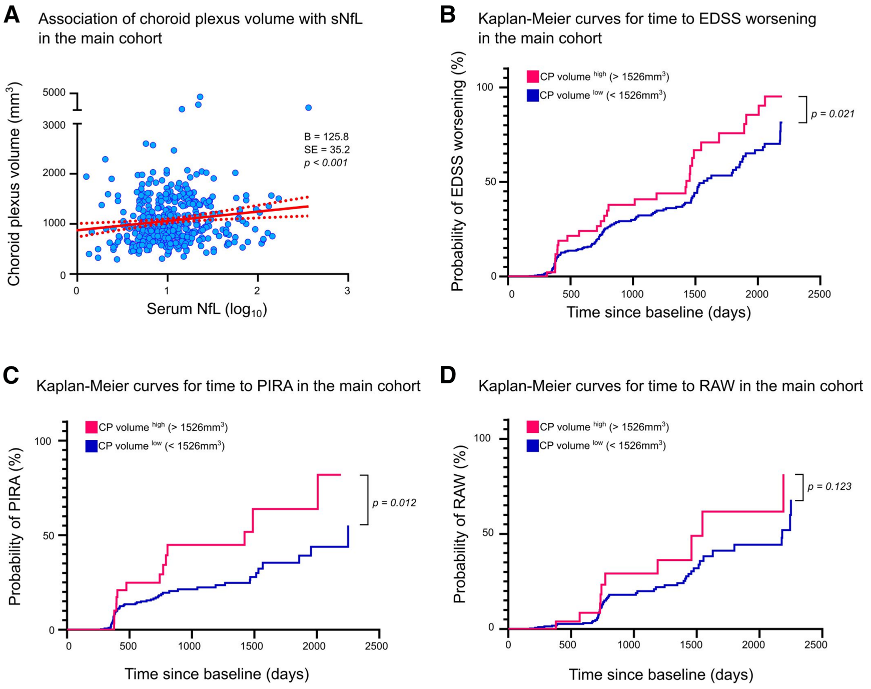

Figure 2. Association of CP volume with sNfL levels and the predictive capacity of CP volume for disability accumulation in MS

(A) Association of choroid plexus (CP) volume with sNfL levels ($\log_{10}$) at baseline in the main cohort. Linear fit (red) to individual values from the main cohort (blue) is shown with 95% CI (dotted red). The regression model was corrected for sex, age, disease duration, Expanded Disability Status Scale (EDSS) scores, disease-modifying treatment, intracranial volume, scanner, lateral ventricle volume, T2 lesion volume, gadolinium-enhancing lesions, and body mass index.

(B–D) Kaplan-Meier curves to predict time to (B) EDSS worsening (CP volume^{low} = 422 and CP volume^{high} = 46), (C) progression independent of relapse activity (PIRA) (CP volume^{low} = 317 and CP volume^{high} = 36), and (D) relapse-associated worsening (RAW) (CP volume^{low} = 300 and CP volume^{high} = 31). Dichotomized groups were based on the optimized cutoff values of CP volume derived from a receiver operating characteristics analysis (STAR Methods). CI, confidence interval; B, regression coefficient; SE, standard error; p, *p* value.

Our findings point toward the prediction of PIRA by a CP volume over 1,526 mm^3 (using FreeSurfer for CP segmentation)—a volume that exceeds the CP size of healthy subjects.[5] In addition, our data indicate that CP volume predicts PIRA independently of sNfL levels. PIRA refers to the amount of accumulated neurological disability occurring independent of relapse activity, a feature that is believed to be linked to non-inflammatory neurodegeneration or compartmentalized inflammation. However, CP enlargement alone does not fully account for the development of PIRA, which likely arises from the interplay of multiple MRI-based markers reflecting both inflammatory and neurodegenerative processes.[14] Based on current evidence, it is difficult to justify a disconnection between inflammatory pathology and neurodegeneration, but the data from our early MS cohort (disease duration less than 5 years) lead us to postulate that the enlarged CP is involved early in mechanisms leading to neuronal loss, demonstrated by high sNfL levels and concomitant relapse-free progression. Although specific pathophysiological mechanisms related to our observed association have not yet been solved, our findings highlight that CP enlargement manifests alongside neurodegenerative processes in MS. Notably, prior studies have reported associations between CP enlargement and both clinical and subclinical inflammatory activity,[5,10,15] including relapses and MRI lesion burden; therefore, we cannot rule out that CP volume may correlate with RAW in other cohorts with higher overt inflammatory activity.

The role of DMTs in modulating CP volume and sNfL levels warrants consideration. High-efficacy DMTs are known to lower sNfL levels.[16] In our regression models, DMT was a significant covariate, yet CP volume remained independently associated

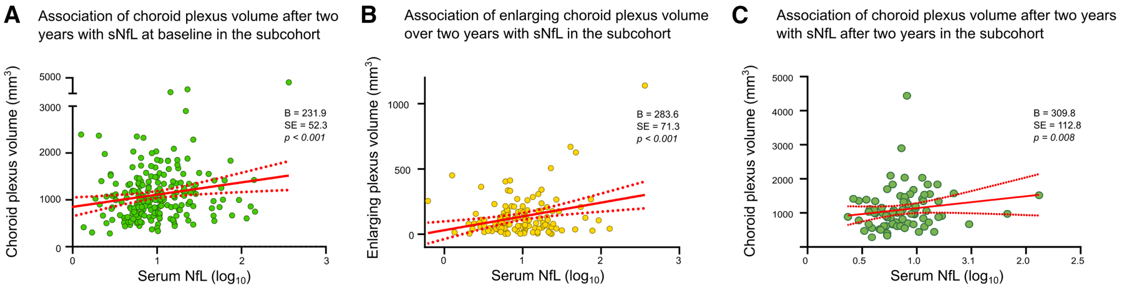

Figure 3. Association of CP volume with sNfL levels in the subcohort with longitudinal MRI

(A) Association of CP volume after 2 years of MRI follow-up with sNfL levels ($\log_{10}$).

(B) Association of enlarging CP volume with sNfL levels ($\log_{10}$).

(C) Association of CP volume after 2 years with sNfL levels ($\log_{10}$) after 2 years.

The regression models were corrected for sex, age, disease duration, Expanded Disability Status Scale (EDSS) scores, disease-modifying treatment, intracranial volume, lateral ventricle volume, number of relapses between MRIs, time between MRIs, T2 lesion volume, gadolinium-enhancing lesions, and BMI. Linear fit (red) to individual values is shown with 95% CI (dotted red). CI, confidence interval; B, regression coefficient; SE, standard error; p, *p* value.

with sNfL levels, supporting a distinct contribution. Although the effect of DMTs on CP morphology remains incompletely understood, evidence suggests that high-efficacy treatment, in particular natalizumab, may modulate immune cell trafficking at the blood-CSF barrier and thereby mitigate CP enlargement.[5]

In this context, potential subtle effects of corticosteroids on CP volume and sNfL levels cannot be entirely excluded in our study, even though we adjusted for clinical relapses (which were largely treated with corticosteroids).

In clinical practice, CP enlargement could support early risk stratification, particularly in identifying pwMS at risk for progression despite the absence of clinical relapses. While its implementation requires further validation and standardization, our findings suggest that CP volume could complement existing imaging and fluid biomarkers in refining individualized treatment decisions.

In conclusion, we provide evidence for the utility of CP imaging as a complementary non-invasive biomarker for neuroaxonal loss already early in the disease and for the development of silent progression over time. CP enlargement predicts neuroaxonal loss in MS, suggesting that structural alterations of the blood-CSF barrier are linked to loss of neuronal integrity and, furthermore, disability worsening independent of acute exacerbations.

Limitations of the study

Our study has some limitations. In humans, we cannot determine whether the relationship between CP enlargement and neuroaxonal loss is causatively linked. CP enlargement in MS is thought to be the result of immune cell aggregates in the CP stroma and vessels, with higher amounts of T lymphocytes, macrophages, and dendritic cells, and changes in the function and structure of ependymal cells including increased permeability of capillaries, thickening of the basement membrane, and loss of cilia in ependymal cells.[4,12,17] Elevated CP volume may reflect structural and functional abnormalities of this physiological barrier associated with an inflammatory state. However, the underlying processes may not only promote acute inflammatory pathology but also neurodegenerative pathology, possibly via smoldering inflammation, in chronic MS.

Another limitation of our study may be the absence of routine spinal cord MRI. While symptomatic spinal relapses were captured and classified as RAW, approximately 12%–15% of spinal cord lesions may be asymptomatic and thus undetected.[18] In addition, EDSS worsening in our study was not formally confirmed after 3 or 6 months as typically performed in clinical trials. However, all instances of EDSS progression (classified as PIRA or RAW) were sustained until the end of follow-up, supporting the robustness of the applied definition and reflecting real-world disease evolution within a large, longitudinal cohort.

Finally, while we applied a standardized imaging protocol across all centers and adjusted for scanner effects by including scanner as a covariate in all models, we did not apply advanced harmonization techniques such as ComBat.[19] Although covariate adjustment is an accepted and effective method when acquisition protocols are uniform (as is the case for our study),[20] residual site effects cannot be entirely excluded.

RESOURCE AVAILABILITY

Lead contact

Further information and requests should be directed to and will be fulfilled by the lead contact, Vinzenz Fleischer (vinzenz.fleischer@unimedizin-mainz.de).

Materials availability

This study did not generate new, unique reagents.

Data and code availability

- Restrictions apply to the availability of these data and are therefore not publicly available. The raw data used in preparation of the figures and tables will be shared in anonymized format upon reasonable request by a qualified investigator for purposes of replicating procedures and results.
- This paper does not report original code.
- Any additional information required to reanalyze the data reported in this paper is available from the lead contact upon request.

ACKNOWLEDGMENTS

The authors thank all study participants as well as Dr. Cheryl Ernest for proofreading and editing the manuscript. The illustrations were adapted from BioRender.com templates (2021). This study was supported by the Deutsche Forschungsgemeinschaft (DFG; SFB CRC-TR-128 [project number 213904703] to F.Z., V.F., and S.B.; SFB 1080 [project number 221828878]; SFB CRC-1292 [project number 318346496] to F.Z.; and SFB/TRR 355 [project number 490846870] to S.B.), the National Multiple Sclerosis Society (NMSS; grant no. RFA-2203-39314, subaward no. GMO 23101, and PO 0000002850 to V.F.), and the Hermann and Lilly Schilling Foundation (to S.B.). The German Competence Network Multiple Sclerosis (KKNMS) was supported by grants from the German Federal Ministry for Education and Research (BMBF).

AUTHOR CONTRIBUTIONS

V.F. and M.S. contributed to data analysis, writing, and figure design. V.F., M.S., G.G.-E., F.S., and T.B. performed data analysis and interpretation. A.S., T.B., F.S., R.S., M.P., N.H., S.G.M., F.L., H.W., L.K., R.G., C.L., S.B., S.G., and F.Z. edited the manuscript for important intellectual content. F.Z. initiated the study together with V.F. All authors had unrestricted access to all data. All authors agreed to submit the manuscript, read and approved the final draft, and take full responsibility for its content.

DECLARATION OF INTERESTS

V.F. has received research support from Novartis. R.S. has received speaker's honoraria from Bayer HealthCare, Alexion Pharma, Novartis Pharma, and Roche Pharma AG; congress travel support from Merck and Biogen Idec GmbH; and research scientific grant support from Novartis Pharma. A.S. has received speaker honoraria from Bristol Myers Squibb, CSL Behring, Merck, Neuraxpharm, and Novartis and research support by the Baasch-Medicus Foundation, the Medical Faculty of the University of Bern, the Swiss MS Society, and the regional association of North Rhine-Westphalia of the German MS Society (DMSG Landesverband NRW), all not related to this work. S.G.M. has received honoraria for lecturing and travel expenses for attending meetings from Almirall, Amicus Therapeutics Germany, Bayer Health Care, Biogen, Bristol Myers Squibb/Celgene, Diamed, Genzyme, MedDay Pharmaceuticals, Merck Serono, Novartis, Novo Nordisk, Ono Pharma, Roche, Sanofi-Aventis, Chugai Pharma, QuintilesIMS, and Teva. His research is funded by the German Ministry for Education and Research, Bundesinstitut für Risikobewertung, Deutsche Forschungsgemeinschaft, Else Kröner Fresenius Foundation, Gemeinsamer Bundesausschuss, German Academic Exchange Service, Hertie Foundation, Interdisciplinary Center for Clinical Research Muenster, German Foundation Neurology, Alexion, Almirall, Amicus Therapeutics Germany, Biogen, Diamed, Fresenius Medical Care, Genzyme, Herz Burgdorf, Merck Serono, Novartis, Ono Pharma, Roche, and Teva. F.L. has received consultancy fees from Roche and support with travel

cost from Teva Pharma. H.W. receives honoraria for acting as a member of Scientific Advisory Boards from Alexion, Argenx, BioCryst, Bristol Meyer Squibb/Celgene, Celerys, Galapagos, Janssen, Merck, Novartis, and Sandoz and speaker honoraria and travel support from Alexion, Biogen, Bristol Meyer Squibb, Genzyme, Merck, Neurodiem, Novartis, Ology, Roche, Teva, and WebMD Global. H.W. is a paid consultant for Actelion, Argenx, Beckton Dickinson, Bristol Meyer Squibb, Dianthus, EMD Serono, Fondazione Cariplo, Gossamer Bio, Idorsia, Immunic, Immunovant, INmune Bio_Syneos Health, Janssen, Lundbeck, LTS, Merck, NexGen, Novartis, Roche, Samsung, Sangamo, Sanofi-Aventis, the Swiss Multiple Sclerosis Society, Toleranzia, UCB Pharma GmbH, Viatris, VirBio, and Worldwide Clinical Trials; his research is funded by DFG, Deutsche Myasthenie Gesellschaft e.V., the European Union, Alexion, Amicus Therapeutics Inc., Argenx, Biogen, CSL Behring, F. Hoffmann-La Roche, Genzyme, Merck, Novartis, Roche, and UCB Pharma GmbH. L.K. has received compensation for serving on Scientific Advisory Boards for Alexion, Biogen, Bristol Myers Squibb, Hexal, Horizon, Janssen, Merck Serono, Novartis, Roche, Sandoz, Sanofi, Teva, and Viatris. She has received speaker honoraria and travel support from Argenx, Bayer, Biogen, Bristol Myers Squibb, Grifols, Horizon, Merck Serono, Novartis, Roche, Sanofi, Santhera, and Teva. She receives research support from the German Research Foundation, the IZKF Münster, Biogen, Novartis, and Merck Serono. R.G. has received compensation for serving as a consultant or speaker from Bayer HealthCare, Biogen Idec, Merck Serono, Novartis, and Teva Neuroscience; he, or the institution he works for, has received research support from Bayer HealthCare, Biogen Idec, Merck Serono, Novartis, and Teva Neuroscience; and he has also received honoraria as a Journal Editor from SAGE and Thieme Verlag. C.L. has received a research grant from the German Federal Ministry for Education and Research (BMBF), German Competence Network Multiple Sclerosis (KKNMS), grant no. 01GI 1601I, and has received consulting and speaker's honoraria from Biogen Idec, Bayer Schering, Daiichi Sankyo, Merck Serono, Novartis, Sanofi, Genzyme, and Teva. S.B. has received honoraria and compensation for travel from Biogen Idec, Merck Serono, Novartis, Sanofi-Genzyme, and Roche. F.Z. has recently received research grants and/or consultation funds from DFG, BMBF, PMSA, MPG, Genzyme, Merck Serono, Roche, Novartis, Sanofi-Aventis, Celgene, ONO, and Octapharma.

STAR★METHODS

Detailed methods are provided in the online version of this paper and include the following:

- KEY RESOURCES TABLE
- EXPERIMENTAL MODEL AND STUDY PARTICIPANT DETAILS
- METHOD DETAILS
  - MRI data acquisition
  - Choroid plexus volume assessment
  - sNfL measurements
- QUANTIFICATION AND STATISTICAL ANALYSIS

SUPPLEMENTAL INFORMATION

Supplemental information can be found online at https://doi.org/10.1016/j.xcrm.2026.102609.

Received: March 31, 2025
Revised: July 11, 2025
Accepted: January 12, 2026
Published: February 9, 2026

REFERENCES

1. Jakimovski, D., Bittner, S., Zivadinov, R., Morrow, S.A., Benedict, R.H., Zipp, F., and Weinstock-Guttman, B. (2024). Multiple sclerosis. Lancet *403*, 183–202. https://doi.org/10.1016/S0140-6736(23)01473-3.
2. Ghersi-Egea, J.F., Strazielle, N., Catala, M., Silva-Vargas, V., Doetsch, F., and Engelhardt, B. (2018). Molecular anatomy and functions of the choroidal blood-cerebrospinal fluid barrier in health and disease. Acta Neuropathol. *135*, 337–361. https://doi.org/10.1007/s00401-018-1807-1.
3. Tan, L.Y., Cunliffe, G., Hogan, M.P., Yeo, X.Y., Oh, C., Jin, B., Kang, J., Park, J., Kwon, M.S., Kim, M., and Jung, S. (2024). Emergence of the brain-border immune niches and their contribution to the development of neurodegenerative diseases. Front. Immunol. *15*, 1380063. https://doi.org/10.3389/fimmu.2024.1380063.
4. Vercellino, M., Votta, B., Condello, C., Piacentino, C., Romagnolo, A., Merola, A., Capello, E., Mancardi, G.L., Mutani, R., Giordana, M.T., and Cavalla, P. (2008). Involvement of the choroid plexus in multiple sclerosis autoimmune inflammation: a neuropathological study. J. Neuroimmunol. *199*, 133–141. https://doi.org/10.1016/j.jneuroim.2008.04.035.
5. Fleischer, V., Gonzalez-Escamilla, G., Ciolac, D., Albrecht, P., Küry, P., Gruchot, J., Dietrich, M., Hecker, C., Müntefering, T., Bock, S., et al. (2021). Translational value of choroid plexus imaging for tracking neuroinflammation in mice and humans. Proc. Natl. Acad. Sci. USA *118*, e2025000118. https://doi.org/10.1073/pnas.2025000118.
6. Klistorner, S., Van der Walt, A., Barnett, M.H., Butzkueven, H., Kolbe, S., Parratt, J., Yiannikas, C., and Klistorner, A. (2023). Choroid plexus volume is enlarged in clinically isolated syndrome patients with optic neuritis. Mult. Scler. *29*, 540–548. https://doi.org/10.1177/13524585231157206.
7. Klistorner, S., Barnett, M.H., Parratt, J., Yiannikas, C., Graham, S.L., and Klistorner, A. (2022). Choroid plexus volume in multiple sclerosis predicts expansion of chronic lesions and brain atrophy. Ann. Clin. Transl. Neurol. *9*, 1528–1537. https://doi.org/10.1002/acn3.51644.
8. Bergsland, N., Dwyer, M.G., Jakimovski, D., Tavazzi, E., Benedict, R.H.B., Weinstock-Guttman, B., and Zivadinov, R. (2023). Association of choroid plexus inflammation on MRI with clinical disability progression over 5 years in patients with multiple sclerosis. Neurology *100*, e911–e920. https://doi.org/10.1212/WNL.0000000000201608.
9. Barro, C., Benkert, P., Disanto, G., Tsagkas, C., Amann, M., Naegelin, Y., Leppert, D., Gobbi, C., Granziera, C., Yaldizli, Ö., et al. (2018). Serum neurofilament as a predictor of disease worsening and brain and spinal cord atrophy in multiple sclerosis. Brain *141*, 2382–2391. https://doi.org/10.1093/brain/awy154.
10. Ricigliano, V.A.G., Louapre, C., Poirion, E., Colombi, A., Yazdan Panah, A., Lazzarotto, A., Morena, E., Martin, E., Bottlaender, M., Bodini, B., et al. (2022). Imaging characteristics of choroid plexuses in presymptomatic multiple sclerosis: a retrospective study. Neurol. Neuroimmunol. Neuroinflamm. *9*, e200026. https://doi.org/10.1212/NXI.0000000000200026.
11. Tsitsou-Kampeli, A., Suzzi, S., and Schwartz, M. (2024). The immune and metabolic milieu of the choroid plexus as a potential target in brain protection. Trends Neurosci. *47*, 573–582. https://doi.org/10.1016/j.tins.2024.05.010.
12. Rodríguez-Lorenzo, S., Konings, J., Van Der Pol, S., Kamermans, A., Amor, S., Van Horssen, J., Witte, M.E., Kooij, G., and De Vries, H.E. (2020). Inflammation of the choroid plexus in progressive multiple sclerosis: accumulation of granulocytes and T cells. Acta Neuropathol. Commun. *8*, 9. https://doi.org/10.1186/s40478-020-0885-1.
13. Alisch, J.S.R., Kiely, M., Triebswetter, C., Alsameen, M.H., Gong, Z., Khattar, N., Egan, J.M., and Bouhrara, M. (2021). Characterization of age-related differences in the human choroid plexus volume, microstructural integrity, and blood perfusion using multiparameter magnetic resonance imaging. Front. Aging Neurosci. *13*, 734992. https://doi.org/10.3389/fnagi.2021.734992.
14. Bsteh, G., Dal-Bianco, A., Krajnc, N., and Berger, T. (2025). Biomarkers of progression independent of relapse activity-can we actually measure it yet? Int. J. Mol. Sci. *26*, 4704. https://doi.org/10.3390/ijms26104704.
15. Ricigliano, V.A.G., Morena, E., Colombi, A., Tonietto, M., Hamzaoui, M., Poirion, E., Bottlaender, M., Gervais, P., Louapre, C., Bodini, B., and Stankoff, B. (2021). Choroid plexus enlargement in inflammatory multiple sclerosis: 3.0-T MRI and translocator protein PET evaluation. Radiology *301*, 166–177. https://doi.org/10.1148/radiol.2021204426.

CellPress
OPEN ACCESS

16. Novakova, L., Zetterberg, H., Sundström, P., Axelsson, M., Khademi, M., Gunnarsson, M., Malmeström, C., Svenningsson, A., Olsson, T., Piehl, F., et al. (2017). Monitoring disease activity in multiple sclerosis using serum neurofilament light protein. Neurology *89*, 2230–2237. https://doi.org/10.1212/WNL.0000000000004683.

17. Preziosa, P., Pagani, E., Meani, A., Storelli, L., Margoni, M., Yudin, Y., Tedone, N., Biondi, D., Rubin, M., Rocca, M.A., and Filippi, M. (2024). Chronic active lesions and larger choroid plexus explain cognition and fatigue in multiple sclerosis. Neurol. Neuroimmunol. Neuroinflamm. *11*, e200205. https://doi.org/10.1212/NXI.0000000000200205.

18. Ostini, C., Bovis, F., Disanto, G., Ripellino, P., Pravatà, E., Sacco, R., Padlina, G., Sormani, M.P., Gobbi, C., and Zecca, C. (2021). Recurrence and prognostic value of asymptomatic spinal cord lesions in multiple sclerosis. J. Clin. Med. *10*, 463. https://doi.org/10.3390/jcm10030463.

19. Fortin, J.P., Cullen, N., Sheline, Y.I., Taylor, W.D., Aselcioglu, I., Cook, P.A., Adams, P., Cooper, C., Fava, M., McGrath, P.J., et al. (2018). Harmonization of cortical thickness measurements across scanners and sites. Neuroimage *167*, 104–120. https://doi.org/10.1016/j.neuroimage.2017.11.024.

20. Pomponio, R., Erus, G., Habes, M., Doshi, J., Srinivasan, D., Mamourian, E., Bashyam, V., Nasrallah, I.M., Satterthwaite, T.D., Fan, Y., et al. (2020). Harmonization of large MRI datasets for the analysis of brain imaging patterns throughout the lifespan. Neuroimage *208*, 116450. https://doi.org/10.1016/j.neuroimage.2019.116450.

21. Lublin, F.D., Häring, D.A., Ganjgahi, H., Ocampo, A., Hatami, F., Čuklina, J., Aarden, P., Dahlke, F., Arnold, D.L., Wiendl, H., et al. (2022). How patients with multiple sclerosis acquire disability. Brain *145*, 3147–3161. https://doi.org/10.1093/brain/awac016.

22. Müller, J., Cagol, A., Lorscheider, J., Tsagkas, C., Benkert, P., Yaldizli, Ö., Kuhle, J., Derfuss, T., Sormani, M.P., Thompson, A., and Granziera, C. (2023). Harmonizing definitions for progression independent of relapse activity in multiple sclerosis: a systematic review. JAMA Neurol. *80*, 1232–1245. https://doi.org/10.1001/jamaneurol.2023.3331.

23. Desikan, R.S., Ségonne, F., Fischl, B., Quinn, B.T., Dickerson, B.C., Blacker, D., Buckner, R.L., Dale, A.M., Maguire, R.P., Hyman, B.T., et al. (2006). An automated labeling system for subdividing the human cerebral cortex on MRI scans into gyral based regions of interest. Neuroimage *31*, 968–980. https://doi.org/10.1016/j.neuroimage.2006.01.021.

24. Fischl, B. (2012). FreeSurfer. Neuroimage *62*, 774–781. https://doi.org/10.1016/j.neuroimage.2012.01.021.

25. Bittner, S., Steffen, F., Uphaus, T., Muthuraman, M., Fleischer, V., Salmen, A., Luessi, F., Berthele, A., Klotz, L., Meuth, S.G., et al. (2020). Clinical implications of serum neurofilament in newly diagnosed MS patients: A longitudinal multicentre cohort study. EBioMedicine *56*, 102807. https://doi.org/10.1016/j.ebiom.2020.102807.

26. Schuckmann, A., Steffen, F., Zipp, F., Bittner, S., and Pape, K. (2023). Impact of extended interval dosing of ocrelizumab on immunoglobulin levels in multiple sclerosis. Med *4*, 361–372.e3. https://doi.org/10.1016/j.medj.2023.05.001.

STAR★METHODS

KEY RESOURCES TABLE

REAGENT or RESOURCE	SOURCE	IDENTIFIER
Software and algorithms		
GraphPad Prism 9.5.1	Graphstats Technologies	https://www.graphpad.com/
BioRender	BioRender	https://www.biorender.com/
FreeSurfer	FreeSurfer	https://surfer.nmr.mgh.harvard.edu/
IBM SPSS Statistics 26	IBM Corporation	https://www.ibm.com/products/spss

EXPERIMENTAL MODEL AND STUDY PARTICIPANT DETAILS

Within this prospective, longitudinal multicenter, pwMS were included over a period of 6 years. All were relapse-free and had received no corticosteroids for at least 30 days prior to enrollment. The study was performed in compliance with the Declaration of Helsinki and was approved by the local ethics committee. All pwMS provided written informed consent. Patients underwent clinical, laboratory and MRI evaluation at baseline. At each follow-up (year 1, 2, 4 and 6), patients were clinically reassessed and EDSS worsening and clinical relapses between visits were collected in order to define PIRA and RAW.[21,22] In this study, PIRA was defined as an increase in EDSS score $\geq$1.0 point from baseline EDSS, occurring in the absence of a clinical relapse between the follow-up visits. RAW was defined as the same EDSS increase occurring in the presence of a documented clinical relapse between the follow-up visits. In this study, only sustained EDSS worsening was considered, meaning that all instances of EDSS increase (whether classified as PIRA or RAW) persisted until the end of the observational period. This approach ensured that transient fluctuations were excluded from progression definitions. A subcohort underwent a subsequent MRI examination at two-year follow-up.

Finally, an additional cohort of participants with secondary progressive MS was identified to examine CP volume across disease stages, given that this phenotype is clinically characterized by a more pronounced neurodegenerative trajectory. This cohort was compared with the individuals with relapsing-remitting MS to investigate CP differences linked to neurodegeneration-driven disability progression (Figure S2).

METHOD DETAILS

MRI data acquisition

Conventional MRI images were acquired at different 3T scanners with a 32-channel receive-only head coil, according to a standardized imaging protocol in all centers. This protocol included sagittal 3D T1-weighted magnetization-prepared rapid gradient echo (MP-RAGE) and T2-weighted fluid-attenuated inversion recovery (FLAIR) sequences. All structural MRI datasets were collected and processed in one analyzing center (Mainz). A detailed description of all acquisition parameters is given in the supplement (Table S1).

Choroid plexus volume assessment

Semi-automated parcellation of CP in the lateral ventricles was performed from T1-weighted images using the open-source FreeSurfer software (Version 6.0),[23,24] followed by visual inspection for quality control and adjustment as appropriate for each subject by an experienced scientist (GGE). FreeSurfer allows segmentation of the cortical and subcortical structures, including the thalamus and CP (Figure S1). To ensure that the results were not driven by other confounding factors, we also computed the intracranial volume and the lateral ventricle volume, and used them as additional independent covariates in the statistical models. Moreover, T2-hyperintense lesion volume and the presence of gadolinium–enhancing lesions were determined and included as covariates in the regression models. Technical details of the volume-based cortical and subcortical parcellation pipeline are described in the supplement.

sNfL measurements

All serum samples were processed according to the standardized biobanking protocol, which ensures harmonized procedures across all participating centers. Serum samples were collected by attending physicians, processed at room temperature within 2 hours, spun at 2000xg for 10 minutes, aliquoted using low-protein-binding polypropylene tubes and low-binding pipette tips to minimize adsorption effects. All aliquots were stored at -80°C until analysis. sNfL concentrations were measured in a blinded fashion as previously described using the highly sensitive single molecule array (SiMoA) technology.[25,26]

QUANTIFICATION AND STATISTICAL ANALYSIS

To evaluate the impact of independent variables on CP volume, multiple regression models were created and adjusted for covariates (see supplemental information). MRI-based covariates were intracranial volume, lateral ventricle volume, T2-hyperintense lesion volume and the presence of gadolinium-enhancing lesions. Scanner/site (i.e., acquisition center) was included as a further categorical covariate in all regression models to account for potential inter-site variability. In a subset of participants with available data, body mass index (BMI) was included as an additional covariate in secondary regression analyses to assess its potential confounding effect. sNfL levels were log-transformed (log10) to ensure linearity. Kaplan-Meier method with Mantel-Cox and Mantel-Haenszel testing was applied to compare time to disability worsening (EDSS worsening, PIRA and RAW) between pwMS with low and high CP volume (excluding those patients with high-efficacy treatment at blood draw). To dichotomize CP volume for prognostication of EDSS worsening, PIRA and RAW receiver operating characteristics (ROC) analyses were performed to identify optimal cut-off values (based on the Youden index). To finally evaluate whether CP volume independently contributes to the risk of disability progression beyond the effects of sNfL levels, a multivariate Cox proportional hazards regression model was performed with CP volume and sNfL levels as covariates. p values < 0.05 were considered significant.

Cell Reports Medicine, Volume *7*

Supplemental information

Choroid plexus enlargement associates with serum neurofilament and predicts relapse-free progression in multiple sclerosis

Vinzenz Fleischer, Muriel Schraad, Gabriel Gonzalez-Escamilla, Ruth Schneider, Tobias Brummer, Falk Steffen, Maria Protopapa, Nicholas Hanuscheck, Anke Salmen, Sven G. Meuth, Felix Luessi, Heinz Wiendl, Luisa Klotz, Ralf Gold, Carsten Lukas, Stefan Bittner, Sergiu Groppa, and Frauke Zipp

SUPPLEMENTAL INFORMATION

Methods S1: MRI preprocessing pipeline and FreeSurfer-based parcellation procedures for CP volume quantification

Detailed acquisition parameters are found in Supplementary Table 1. Before pre-processing, the nonparametric non-uniform normalization correction was applied to all images to reduce intensity non-uniformity. For all participants, semi-automated parcellation of the choroid plexus (CP) in the lateral ventricles was performed from T1-weighted images using FreeSurfer (version 6.0; https://surfer.nmr.mgh.harvard.edu/). To ensure the robustness of CP volume measurements, a subset of scans was independently re-segmented by two raters, and visual inspection confirmed a high degree of consistency between segmentations, with no systematic deviations observed. Briefly, a probabilistic atlas, built by manual labeling on a training dataset normalized to the MNI305 space resulting in a point-to-point correspondence between all training subjects, is used as parcellation prior for all brain regions [1,2]. This atlas provides the probability of each brain region to belong to a given voxel, the probability of each brain region given the classification of neighboring voxels (neighborhood function), and the probability distribution function of voxel intensities, modelled as a normal distribution, for each brain region at each voxel. Then, newly introduced images are parcelled by normalizing the new image to the common space and incorporating the subject-specific voxel intensities to find the optimal parcellation that maximizes the probability of observing the input data. To ensure that the results including CP volumes are not driven by other confounding factors, we also extracted total intracranial volume and lateral ventricle volume. Following brain CP and ventricle parcellation by tiling the boundary of white matter mass, an initial white surface is created for each cerebral hemisphere, which is further refined following intensity gradients of the white and gray matter to generate the final gray–white surface. This surface is then extended to follow the intensity gradient of gray matter and cerebrospinal fluid (CSF), leading to the creation of the pial surface. Finally, cortical thickness at each surface vertex is computed as the average distance from each vertex in the gray–white surface to the corresponding point in the pial surface [1,2].

Supplementary Figure S1. Association of CP volume and MRI-derived atrophy measures. Related to STAR Methods and the Results. (A) Association between CP volume and cortical thickness in pwMS (sex- and age-adjusted). **(B)** Association between CP volume change and cortical thickness change over two years (sex- and age-adjusted). **(C)** Association between CP volume and thalamic volume (sex- and age-adjusted). **(D)** Association between CP volume change and thalamic volume change (atrophy) over two years (sex- and age-adjusted). **(E)** Association between CP volume and subcortical grey matter (GM) volume (sex- and age-adjusted). **(F)** Association between CP volume change and subcortical GM volume change (atrophy) over two years (sex- and age-adjusted). Linear fits (red solid lines) to individual data points are shown with 95% confidence intervals (dotted lines).

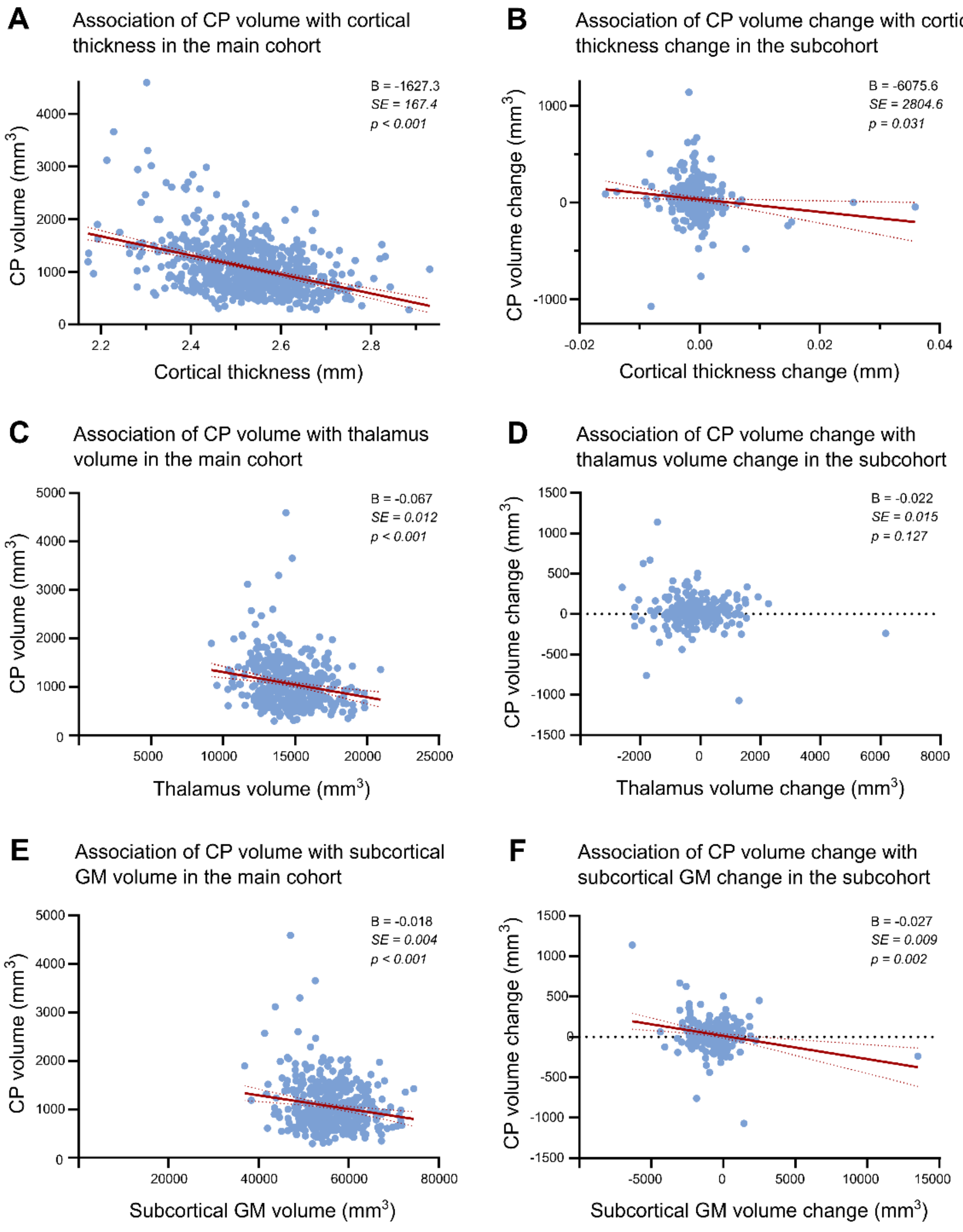

Supplementary Figure S2. CP enlargement in relapsing-remitting MS (RRMS) compared to a cohort with secondary progressive MS (SPMS). Related to STAR Methods and the Results. (A) Comparison of absolute CP volume between people with RRMS and SPMS (n=90), showing significantly higher CP volumes in the SPMS group. **(B)** Comparison of CP volume normalized to age (CP volume/age) between RRMS and SPMS, demonstrating that the difference remains significant after age correction. Violin plots depict the full data distribution with median values indicated by horizontal lines.

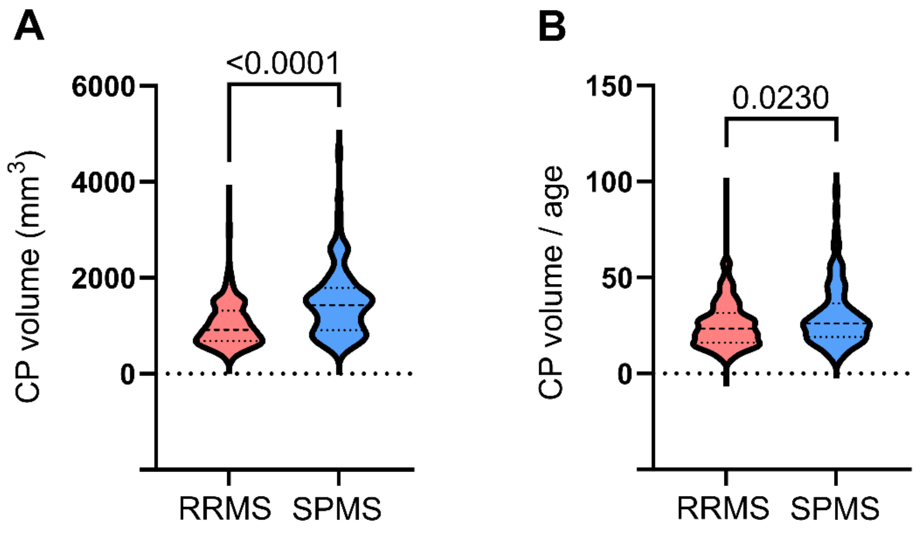

Supplementary Table 1. MR scanners and image acquisition parameters of the centers. Related to STAR Methods.

Center	Scanner (vendor)	No. of patients	3D T1-weighted sequence	3D T2-weighted FLAIR sequence
Mainz	Magnetom Trio (Siemens)	274	192 sagittal slices with 1 mm isotropic voxel size and a 256 x 256 matrix size TE/TI/TR = 2.52/900/1900 ms	192 sagittal slices with 1 mm isotropic voxel size and a 256 x 256 matrix size TE/TI/TR = 388/1800/5000 ms
Münster	Magnetom Prisma (Siemens)	87	192 sagittal slices with 1 mm isotropic voxel size and a 256 x 256 matrix size TE/TI/TR = 2.2/900/2130 ms	192 sagittal slices with 1 mm isotropic voxel size and a 256 x 256 matrix size TE/TI/TR = 389/1800/5000 ms
Bochum	Achieva (Philips)	71	180 sagittal slices with 1 mm isotropic voxel size and a 256 x 256 matrix size TE/TI/TR = 4.6/1000/10 ms	170 sagittal slices with 1 mm isotropic voxel size and 256 x 256 matrix size TE/TI/TR = 286/1650/4800 ms

Supplementary Table 2. Regression model on MRI-derived CP volume at baseline. Related to Figure 2A. Linear regression model of covariables influencing the association between CP volume and sNfL levels in pwMS who underwent baseline MRI (n=434). The regression model was corrected for sex, age, disease duration, Expanded Disability Status Scale (EDSS) score, disease-modifying treatment (DMT), intracranial volume, scanner and lateral ventricle volume. T2 lesion volume (n=326) was inserted into the regression model; the presence of gadolinium (Gad)-enhancing lesions (n=276) and body mass index (BMI; n=177) were subsequently inserted into that regression model. Regression coefficients B with 95% confidence interval (lower and upper bound) and level of significance are given. P values <0.05 were considered significant.

Independent variables	Regression coefficient B	p-value	Lower bound 95%CI	Upper bound 95%CI
**Sex**	-49.719	*0.119*	-112.213	12.775
**Age**	3.741	*0.003*	1.288	6.193
**Disease duration**	0.482	*0.284*	-0.402	1.366
**EDSS**	-5.000	*0.676*	-28.536	18.536
**DMT**	108.502	*0.073*	-10.291	227.296
**Intracranial volume**	0.000	*0.263*	0.000	0.000
**Scanner**	-38.742	*0.195*	-97.352	19.868
**Lateral ventricle volume**	0.033	*<0.001*	0.030	0.035
**sNfL (log_{10})**	125.769	*<0.001*	56.573	194.965
**T2 lesion volume**	4.929	*<0.001*	2.627	7.231
**Gad-enhancing lesions**	49.878	*0.173*	-22.039	121.796
**BMI**	-5.834	*0.093*	-12.648	0.979

Supplementary Table 3. Regression model on MRI-derived CP volume after two years. Related to Figure 3A. Linear regression model of covariables influencing the association between the change in CP volume and sNfL levels in pwMS who underwent baseline and 2-year follow-up MRI (n=208). The regression model was corrected for sex, age, disease duration, Expanded Disability Status Scale (EDSS) score, disease-modifying treatment (DMT), intracranial volume, lateral ventricle volume, number of relapses between the MRIs and time between the MRIs. T2 lesion volume at baseline (n=208) was inserted into the regression model; the presence of gadolinium (Gad)-enhancing lesions at baseline (n=208) and body mass index (BMI; n=70) were subsequently inserted into that regression model. Regression coefficients B with 95% confidence interval (lower and upper bound) and level of significance are given. P values <0.05 were considered significant.

Independent variables	Regression coefficient B	p-value	Lower bound 95%CI	Upper bound 95%CI
**Sex**	-183.815	*0.002*	-297.521	-70.109
**Age**	3.208	*0.101*	-0.627	7.043
**Disease duration**	-0.511	*0.323*	-1.526	0.505
**EDSS**	-4.509	*0.779*	-36.174	27.155
**DMT**	58.422	*0.292*	-50.555	167.400
**Intracranial volume**	-0.001	*0.021*	-0.003	-0.001
**Lateral ventricle volume**	0.038	*<0.001*	0.034	0.042
**No. of relapses between MRIs**	9.586	*0.676*	-35.527	54.700
**Time between MRIs**	0.920	*0.502*	-1.779	3.619
**sNfL (log_{10})**	231.904	*<0.001*	128.815	334.993
**T2 lesion volume**	7.485	*0.005*	2.338	12.632
**Gad-enhancing lesions**	60.739	*0.228*	-38.25	159.729
**BMI**	-5.019	*0.489*	-19.442	9.403

Supplementary Table 4. Regression model on MRI-derived CP volume change over two years. Related to Figure 3B. Linear regression model of covariables influencing the association between the change in CP volume in pwMS who underwent baseline and 2-year follow-up MRI and displayed CP enlargement (n=122). The regression model was corrected for sex, age, disease duration, Expanded Disability Status Scale (EDSS) change, disease-modifying treatment (DMT), intracranial volume, lateral ventricle volume, number of relapses between the MRIs and time between the MRIs. T2 lesion volume at baseline (n=122) was inserted into the regression model; the presence of gadolinium (Gad)-enhancing lesions at baseline (n=122) and body mass index (BMI; n=43) were subsequently inserted into that regression model. Regression coefficients B with 95% confidence interval (lower and upper bound) and level of significance are given. P values <0.05 were considered significant.

**Independent variables**	**Regression coefficient B**	**p-value**	**Lower bound 95%CI**	**Upper bound 95%CI**
**Sex**	-161.013	*0.072*	-336.854	14.829
**Age**	5.398	*0.069*	-0.431	11.226
**Disease duration**	-0.614	*0.503*	-2.421	1.194
**EDSS change**	-34.868	*0.151*	-82.645	12.909
**DMT**	67.711	*0.400*	-91.23	226.652
**Intracranial volume**	-0.001	*0.048*	-0.002	-0.001
**Lateral ventricle volume**	0.042	*<0.001*	0.035	0.048
**No. of relapses between MRIs**	53.98	*0.164*	-22.388	130.348
**Time between MRIs**	0.412	*0.841*	-3.639	4.463
**sNfL (log_{10})**	283.627	*<0.001*	142.322	424.932
**T2 lesion volume**	13.041	*0.003*	4.456	21.625
**Gad-enhancing lesions**	21.008	*0.754*	-111.467	153.482
**BMI**	-3.622	*0.727*	-3.622	-3.622

Supplementary Table 5. Regression model on MRI-derived CP volume after two years. Related to Figure 3C. Linear regression model of covariables influencing the association between the CP volume and sNfL levels in pwMS who underwent 2-year follow-up MRI and sNfL measurement (n=81). The regression model was corrected for sex, age, disease duration, Expanded Disability Status Scale (EDSS) score, disease-modifying treatment (DMT), intracranial volume, lateral ventricle volume, and T2 lesion volume at 2 years. Regression coefficients B with 95% confidence interval (lower and upper bound) and level of significance are given. P values <0.05 were considered significant.

Independent variables	Regression coefficient B	p-value	Lower bound 95%CI	Upper bound 95%CI
**Sex**	-235.816	*0.005*	-397.260	-74.372
**Age**	-0.891	*0.709*	-5.631	3.849
**Disease duration**	-5.496	*0.000*	-7.710	-3.282
**EDSS**	-28.656	*0.201*	-72.904	15.592
**DMT**	85.883	*0.192*	-44.043	215.810
**Intracranial volume**	-0.001	*0.048*	-0.002	-0.001
**Lateral ventricle volume**	0.042	*<0.001*	0.036	0.047
**sNfL (log_{10})**	309.798	*0.008*	84.871	534.726
**T2 lesion volume**	7.210	*0.008*	1.908	12.512

References

1. Fischl, B. (2012). FreeSurfer. Neuroimage *62*, 774-781. 10.1016/j.neuroimage.2012.01.021.
2. Desikan, R.S., Segonne, F., Fischl, B., Quinn, B.T., Dickerson, B.C., Blacker, D., Buckner, R.L., Dale, A.M., Maguire, R.P., Hyman, B.T., et al. (2006). An automated labeling system for subdividing the human cerebral cortex on MRI scans into gyral based regions of interest. Neuroimage *31*, 968-980. 10.1016/j.neuroimage.2006.01.021.
